# Galectin-3 is elevated in CSF and is associated with Aβ deposits and tau aggregates in brain tissue in Alzheimer’s disease

**DOI:** 10.1007/s00401-022-02469-6

**Published:** 2022-07-27

**Authors:** Antonio Boza-Serrano, Agathe Vrillon, Karolina Minta, Agnes Paulus, Lluís Camprubí-Ferrer, Megg Garcia, Ulf Andreasson, Anna Antonell, Malin Wennström, Gunnar Gouras, Julien Dumurgier, Emmanuel Cognat, Laura Molina-Porcel, Mircea Balasa, Javier Vitorica, Raquel Sánchez-Valle, Claire Paquet, Jose Luis Venero, Kaj Blennow, Tomas Deierborg

**Affiliations:** 1 Experimental Neuroinflammation Laboratory, Department of Experimental Medical Science, Lund University, 22184 Lund, Sweden; 2 Alzheimer’s Disease and Other Cognitive Disorders Unit, Neurology Service, Hospital Clínic de Barcelona, IDIBAPS, Universitat de Barcelona, Barcelona, Spain; 3 Department of Biochemistry and Molecular Biology, Faculty of Pharmacy, University of Seville and IBIS (Institute of Biomedicine of Seville), University Hospital Virgen del Rocio, CSIC, Seville, Spain; 4 Université Paris Cité, Inserm U1144, Paris, France; 5 Center of Cognitive Neurology, Université Paris Cité, Lariboisière Fernand-Widal Hospital, APHP, Paris, France; 6 Department of Psychiatry and Neurochemistry, The Sahlgrenska Academy at the University of Gothenburg, Mölndal, Sweden; 7 Medical Microspectroscopy Laboratory, Department of Experimental Medical Science, Lund University, 22184 Lund, Sweden; 8 Clinical Neurochemistry Laboratory, Sahlgrenska University Hospital, Mölndal, Sweden; 9 Neurological Tissue Bank, Biobanc-Hospital Clínic-IDIBAPS, Barcelona, Spain; 10 Clinical Memory Research Unit, Department of Clinical Sciences Malmö, Lund University, Malmö, Sweden; 11 Experimental Dementia Laboratory, Department of Experimental Medical Sciences, Lund University, 221 84 Lund, Sweden; 12 Centro de Investigación Biomedica en Red Sobre Enfermedades Neurodegenerativas (CIBERNED), Madrid, Spain

## Abstract

Galectin-3 (Gal-3) is a beta-galactosidase binding protein involved in microglial activation in the central nervous system (CNS). We previously demonstrated the crucial deleterious role of Gal-3 in microglial activation in Alzheimer’s disease (AD). Under AD conditions, Gal-3 is primarily expressed by microglial cells clustered around Aβ plaques in both human and mouse brain, and knocking out Gal-3 reduces AD pathology in AD-model mice. To further unravel the importance of Gal-3-associated inflammation in AD, we aimed to investigate the Gal-3 inflammatory response in the AD continuum. First, we measured Gal-3 levels in neocortical and hippocampal tissue from early-onset AD patients, including genetic and sporadic cases. We found that Gal-3 levels were significantly higher in both cortex and hippocampus in AD subjects. Immunohistochemistry revealed that Gal-3+ microglial cells were associated with amyloid plaques of a larger size and more irregular shape and with neurons containing tau-inclusions. We then analyzed the levels of Gal-3 in cerebrospinal fluid (CSF) from AD patients (*n* = 119) compared to control individuals (*n* = 36). CSF Gal-3 levels were elevated in AD patients compared to controls and more strongly correlated with tau (p-Tau181 and t-tau) and synaptic markers (GAP-43 and neurogranin) than with amyloid-β. Lastly, principal component analysis (PCA) of AD biomarkers revealed that CSF Gal-3 clustered and associated with other CSF neuroinflammatory markers, including sTREM-2, GFAP, and YKL-40. This neuroinflammatory component was more highly expressed in the CSF from amyloid-β positive (A+), CSF p-Tau181 positive (T+), and biomarker neurodegeneration positive/negative (N+/−) (A + T + N+/−) groups compared to the A + T−N− group. Overall, Gal-3 stands out as a key pathological biomarker of AD pathology that is measurable in CSF and, therefore, a potential target for disease-modifying therapies involving the neuroinflammatory response.

**Supplementary Information:**

The online version contains supplementary material available at 10.1007/s00401-022-02469-6.

## Introduction

Alzheimer’s disease (AD) is a neurodegenerative disorder characterized by the formation of amyloid-β (Aβ) deposits and intraneuronal tau aggregates called neurofibrillary tangles. Recent transcriptomic data highlight a critical role of the innate immune system in AD pathology [28–30]. Genome-wide association studies have also identified innate immunity-related variants in genes such as *TREM-2*, *CD33*, *CR1* and *MEF2C *[14, 15, 20, 56] that are associated with a significant risk of developing AD. Microglial cells are central nervous system (CNS) resident macrophages. They play diverse roles, including brain parenchyma surveillance, phagocytosis, synaptic remodeling, and inflammatory response [11]. Indeed, reactive microglial cells surrounding Aβ plaques are a pathological hallmark of AD. A specific AD microglial phenotype was recently discovered, the so-called disease-associated microglia (DAM) [2]. DAM is characterized by the upregulation of *TREM-2*, *ApoE*, *Spp1*, *Itgax* and *Axl* and seems to play a beneficial role in AD [29]. On the other hand, a more detrimental microglial subtype has recently been characterized, the so-called microglia neurodegenerative (MGnD) [30]. This phenotype is associated, among others, with *Cecl7a*, *Lgals3*, *TREM2* and *Ccl2* gene upregulation*.*

Galectin-3 (Gal-3) is a beta-galactosidase binding protein encoded by *LGALS3* and is mainly expressed by microglial cells in the CNS. Gal-3 consists of one N-terminal domain and one carbohydrate recognition domain [48] and is released into the extracellular space by activated microglia. Once in the extracellular space, Gal-3 can act autocrine or paracrine by binding to different membrane receptors, such as TLR4 and TREM-2 [8, 10]. Previously, we demonstrated that Gal-3 plays a detrimental role in microglial activation in AD [8]. First, we found that Gal-3 was highly upregulated in cortical tissue from AD patients compared to age-matched controls. There, Gal-3 was found primarily in microglia clustered around Aβ plaques. Next, we generated a mouse model based on the 5xFAD model of AD but lacking Gal-3. The lack of Gal-3 in 5xFAD mice lessened the Aβ burden and improved cognitive performance [8]. Our study also confirmed that Gal-3 was linked to TREM-2 activity by employing STORM microscopy, fluorescence anisotropy, and a TREM-2–DAP12 reporter cell line. The role of Gal-3 in AD was later supported by Chih-Chieh Tao et al. [53], who found that Gal-3 is involved in disease progression and Aβ oligomerization using amyloid precursor protein (APP) transgenic mice lacking Gal-3.

Further, a recent large-scale proteomic analysis using human AD brain tissue (> 2000 brains) highlighted a microglia module as one of the most affected processes in AD brain. An astrocytic/microglial metabolism module was significantly enriched in gene products connected to AD risk factor loci [28]. The study pointed out the top 30 most differentially abundant microglial transcripts in an AD mouse model that correspond with proteins in the human microglia module related to AD pathology. Remarkably, within this list, Gal3 ranked fifth, supporting its role as one of the key molecules related to AD pathology [28].

Cerebrospinal fluid (CSF) plays a key role in brain metabolism and can be used to measure the concentration of pathological hallmarks related to AD pathology progression [4, 25, 46]. So far, measures of the Aβ42/Aβ40 ratio, total tau (t-tau) and hyperphosphorylated tau (p-Tau) are the most reliable markers for disease diagnosis. As the AD process initiates, CSF Aβ42 levels drop and the levels of different isoforms of tau increase, which is associated with neurodegeneration [4]. Similarly, inflammatory molecules can be detected in the CSF of AD patients [37]. For instance, markers of astrocytic activation, namely GFAP and YKL-40, are elevated in the CSF of AD patients and linked to pathology progression [2, 5, 24]. A key microglial marker, TREM-2, has also been noted as being elevated in AD patient CSF [18, 51] and has recently been linked to slower Aβ deposition [17], reduced cognitive decline [18] and tau-related neurodegeneration [52]. Interestingly, Gal-3 has been detected in the CSF of AD patients and its measures suggest that it could be elevated in AD [21, 59].

Considering our previous findings on the role of Gal-3 in AD progression and the interaction between Gal-3 and TREM-2, we: (i) further explored Gal-3 expression in AD brain and (ii) explored whether CSF Gal-3 levels correlate with levels of Aβ, inflammatory markers, including TREM-2, and neurodegenerative biomarkers in CSF in AD. First, we studied Gal-3 levels in frontal cortex and hippocampal tissue from genetic and sporadic AD cases from the Neurological Tissue Bank of the Hospital Clinic de Barcelona-IDIBAPS. We wanted to expand our previous findings by measuring Gal-3 levels in different brain areas and different AD phenotypes. We could determine whether AD-related genetic mutations alter Gal-3 microglial expression in AD by comparing genetic cases with sporadic cases. To further elucidate the role of Gal-3-dependent microglial activation in AD, we performed immunohistochemistry on human brain sections from AD patients. We also analyzed the association between Gal-3, amyloid plaques and tau neurofibrillary tangles.

The second main goal of this study was to determine whether Gal-3 in CSF could be an AD biomarker. Therefore, we studied CSF Gal-3 levels in patients with evidence of AD physiopathology compared to control subjects. In CSF, we evaluated the association between Gal-3 and the main pathological hallmarks of the disease, Aβ and Tau. Moreover, we studied the relationship between levels of Gal-3 and two synaptic markers, Neurogranin and GAP-43, in addition to inflammatory markers, including GFAP and TREM-2. In addition, we evaluated whether Gal-3 levels were linked to cognitive decline in AD. Lastly, principal component analysis (PCA) was performed to determine whether we could detect distinct populations in the cohort defined by biomarker composition, i.e., inflammation vs. classic AD biomarkers.

## Materials and methods

### CSF cohort

A total of 155 participants were recruited from the Cognitive Neurology Center, APHP Université de Paris Lariboisière Fernand-Widal Hospital in Paris, France, including *n* = 36 neurological controls (NC) and *n* = 119 AD patients. All patients who had undergone a lumbar puncture to explore a cognitive complaint had a CSF sample collected at the BioCogBank Lariboisière Paris. Patients underwent a comprehensive neurological examination, neuropsychological assessment, morphological brain imaging and lumbar puncture. AD diagnosis was made according to the NIA-AAA’s most recent diagnostic research criteria [25]. CSF profile was classified according to the ATN classification: A+/− indicating biomarker evidence of Aβ deposition; T ± indicating biomarker evidence of pathologic tau; and N+/− indicating biomarker evidence of neurodegeneration [26]. CSF biomarker profiles of AD patients fell onto a continuum defined by decreasing Aβ_42/40_ ratio. NC had normative or sub-normative cognitive scores, normal brain morphology and a normal CSF biomarker profile (A−T−N−). Included subjects gave informed consent to participate, and the study was approved by the Bichat Ethics Committee (*n*°10-037 18/03/2010) and followed the principles of the Declaration of Helsinki. Demographic data from the CSF cohorts is described in Table 1.

**Table 1:** Demographics and biomarkers values

*N* = 155	Neurological controls *N* = 36	AD *N* = 119	*P* value
Female sex	72% (26)	60% (72)	0.140
Age	62.7 (9.67)	72.4 (8.0)	< 0.001
LoE	12.48 (3.472)	11.4 (3.6)	0.060
ApoE4 carriership	25% (9)	60% (72)	< 0.001
MMSE	27. 0 (2.1)	21.2 (5.5)	< 0.001
CSF markers			
Aβ42, pg/mL	1059.3 (311.5)	557.9 (299.1)	< 0.001
Aβ42/Aß40 ratio	0.099 (0.034)	0.044 (0.011)	< 0.001
CSF t-tau, pg/mL	223.6 (65.9)	667.7 (350.4)	< 0.001
CSF p-Tau, pg/mL	32.9 (8.1)	104.3 (55.8)	< 0.001
CSF GAL-3, pg/mL	960.5 (177.4)	1168.8 (332.2)	0.037
CSF sTREM-2, pg/mL	3523.7 (1411.7)	4582.0 (1924.4)	0.124
CSF YKL-40, pg/mL	1625.5 (632.3)	2392.9 (2230.4)	0.034
CSF GFAP, pg/mL	1913.3 (1247.3)	4633.0 (3660.2)	< 0.001
CSF neurogranin, pg/mL	144.9 (73.4)	259.2 (82.4)	< 0.001
CSF GAP-43, pg/mL	2416.4 (706.2)	4306.9 (1880.1)	< 0.001

### Human brain tissue

Frozen hippocampal and cerebral cortical tissue from non-demented controls (*n* = 10), EOAD cases (*n* = 11) and genetic AD cases (*n* = 9) (Neurological Tissue Bank, Biobanc-Hospital Clínic-IDIBAPS, Barcelona, Spain) and sections of formalin-fixed and glucose-immersed hippocampal tissue from non-demented controls (*n* = 3) and AD cases (*n* = 8) (The Netherlands Brain Bank) were analyzed. Detailed neurological assessment of the samples is available in Supp. Table 1 (online resources). Written informed consent for the use of brain tissue and clinical data for research purposes was obtained from all patients or their next of kin following the International Declaration of Helsinki and Europe’s Code of Conduct for Brain Banking. The medical ethics committee of VU medical center in Amsterdam and the IRB of Clinic Hospital in Barcelona approved the procedures for brain tissue collection. The regional ethical review board in Sweden approved the study.

### Protein extraction

RIPA solution was prepared with a protease inhibitor (cOmplete Protease Inhibitor Cocktail, Roche) and phosphatase inhibitor (PhoSStop, Roche). Frozen human tissue samples of hippocampus and cerebral cortex were homogenized in RIPA buffer (1 mL/100 μg of tissue, Sigma-Aldrich, Germany) and briefly sonicated. The pellet was subsequently ultracentrifuged at 25,000*g* for 25 min. The supernatant was later used for analysis.

### Western blotting

Protein extracts in RIPA were separated by SDS-PAGE using pre-cast gels (4–20%, Bio-Rad) in Tris–Glycine-SDS buffer (Bio-Rad, Spain). Protein was transferred to nitrocellulose membranes (Bio-Rad) using the TransBlot Turbo system from Bio-Rad. Membranes were subsequently blocked for 1 h with skim milk at 5% (w/v) in PBS and then washed three times for 10 min each in PBS supplemented with 0.1% (v/v) Tween 20 (PBS-T). Membranes were then incubated with primary antibodies in PBS-T overnight. Following this, the membranes were incubated with secondary antibodies for 2 h. After the secondary antibody incubation, membranes were washed three times with PBS-T. According to the manufacturer’s protocol, the membranes were developed using SuperSignal West Pico PLUS Chemiluminescent Substrate (ThermoScientific, Spain) and imaged using a ImageQuant LAS-4000 biomolecular imager (GE Healthcare).

### MSD ELISA

Meso Scale Discovery (MSD) kits were used to measure the levels of Aβ42, p-Tau, and t-tau in the RIPA fraction of the human brain. Serial dilutions of the RIPA fractions were tested to measure protein levels accurately. Before performing the assay, protein levels were measured using a Thermofisher BCA Assay Kit. With those results, 1 μg of protein from the soluble fraction was diluted to evaluate Aβ42, t-tau and p-Tau levels. The plates were developed and read using a QuickPlex Q120 reader (Meso Scale Diagnostics). ELISA plates from Abcam (ab269555) were used to measure the levels of Gal-3 (detection range 58.8–2000 pg/ml) in tissue homogenates. The protocol was carried out according to the manufacturer’s instructions. A Biotek Synergy 2 was used to read the ELISA Gal-3 assay.

### Immunohistochemistry

Hippocampal sections 40-µm-thick were washed (3 × 15 min) in 0.1 M KPBS and then incubated in 0.1 M KPBS, Tween 20 0.25% and normal donkey serum 5% for one hour at room temperature. For immunofluorescence labeling (Iba1, Gal-3, Aβ, or p-Tau), sections were first incubated with the primary antibodies followed by the corresponding Alexa 488/555/647 secondary antibodies (1:1000 dilution, AlexaFluor, Life Technologies). After mounting and drying on slides, the sections were incubated in 0.6 g Sudan Black (Sigma) dissolved in 70% ethanol for 5 min. Subsequently, the sections were washed in PBS and mounted with the mounting medium. When imaging, the camera settings were adjusted at the start of the experiment and maintained for uniformity. A Nikon Eclipse Ti confocal microscope (Nikon, Japan) and NIS elements software (Nikon, Japan) were used to take 20 × magnification pictures and for the final collage. Primary antibodies included anti-Iba1 (Wako, 019-19741), Gal-3 (R&D, AF1197), Aβ (6E10, Covance), and p-Tau (Thermofisher, MN120). NIS Element Analysis software (Nikon, Japan) was used to evaluate plaque size and shape. To create the 3D model, we used Imaris version 9.8 (Oxford Instruments). For the model, image stacks were taken using a Leica TCS SP8 laser scanning confocal microscope (Leica Microsystems) with the Leica Application Suite X software version 3.5.7 (Leica Microsystems).

### CSF biomarker analysis

Lumbar puncture was performed after overnight fasting. CSF samples were centrifuged at 1000 × *g* for 10 min at 4 °C within 2 h of collection and then aliquoted into 0.5 mL polypropylene tubes before being stored at − 80 °C for further analysis. All biomarkers assessed in this study were measured at the Clinical Neurochemistry Laboratory at the University of Gothenburg in Mölndal, Sweden. Gal-3 was measured in CSF using commercially available sandwich ELISA kits (Abcam ab269555, Cambridge, UK) following the manufacturer’s instructions. Samples were analyzed in singlicate with standards run in duplicate. Duplicates of CSF pool quality controls were placed at the beginning and end of each plate. In a polystyrene 96-well plate, pre-diluted 1:2 CSF samples, CSF pool quality controls and standards were incubated with a solution containing an affinity tag-labeled capture Gal-3 antibody and a reporter-conjugated detection Gal-3 antibody. The entire sandwich complex was immobilized to the well via immunoaffinity of an anti-tag antibody. Following the washing procedure, wells were incubated with 3,3′,5,5′-Tetramethylbenzidine TMB. The addition of a stop solution terminated the reaction, and the absorbance was read in a Sunrise microplate absorbance reader (Tecan, Männedorf, Switzerland) at 450 nm. Four-parameter logistic regression was used for standard curve-fitting analysis. All samples were within detection range and were quantified. The sensibility indicated by the manufacturer was 13.3 pg/mL. The coefficients of variation (CVs) for intra- and inter-plate variations were 4.9% and 5.9%, respectively.

CSF soluble TREM-2 (sTREM-2) was measured in-house using an electrochemiluminescence immunoassay with a Meso Scale Discovery (MSD) SECTOR imager 6000 (MSD, Rockville, MD), as previously described by Alosco et al. [1]. In brief, the capture antibody was a biotinylated polyclonal goat anti-human TREM-2 antibody (0.25 μg/mL R&D Systems, Minneapolis, MN), and the detector antibody was a monoclonal mouse anti-human TREM-2 antibody (1 μg/Ml Santa Cruz Biotechnology, Dallas, TX). Recombinant human TREM-2 (4000–62.5 pg/mL) was used to compute a calibration curve. Samples were diluted 1:4 for analysis. The intra- and inter-plate CVs were 3.8% and 4.9%, respectively. CSF levels of Aβ42, Aβ40, p-Tau181 and t-tau were measured with the commercially available LUMIPULSE G1200 automated immunoassay instrument following the manufacturer’s instructions (Fujirebio). Diagnostic cutoffs used were the following: Aß42/Aß40 < 0.61 pg/mL; p-Tau181 < 61 pg/mL; t-tau < 450 pg/mL. CSF neurofilament light (NfL) was measured using an in-house sandwich ELISA with capture and detection antibodies directed against the central rod domain of the protein, NfL21 and NfL23, respectively, as described previously [19]. The intra- and inter-plate variation CVs were 6.7% and 8.4%, respectively. CSF YKL-40 was measured using a commercially available ELISA kit (R&D Systems, Minneapolis, MN). The intra-plate CV was 8.7%, and the inter-plate CV was 10.8%. CSF GFAP was quantified using the HD-X SIMOA platform using a commercial kit by Quanterix (Billerica, MA, USA). The intra-plate CV was 6.1%, and the inter-plate CV was 6.0%.

### Statistical analysis

Statistical analysis was carried out using GraphPad Prism version 9 (GraphPad Software, San Diego, CA, USA) and SPSS v. 26 (IBM Corp., Armonk, NY, USA) software. Normality was assessed by Kolmogorov–Smirnov test. All AD-related variables from the cortical and hippocampal tissue were analyzed with a non-parametric test. Mann–Whitney test was used to compare independent groups. For multiple comparisons, the Kruskal–Wallis test was performed followed by Dunn’s post hoc correction. Chi-square (*χ*^2^) tests were used to investigate the differences between groups with categorical variables. Outlier analysis was performed in GraphPad to identify anomalous observations in the dataset. Brain tissue samples from patients with acute or subacute infarct were excluded from the analysis. CSF biomarker levels were compared between AD and NC with linear regression adjusted for age, sex and ApoE4 carriership. The correlation of Gal-3 with other biomarkers values was analyzed using Spearman’s rank correlation. We calculated the area under receiver-operating characteristic curve (AUROC) to study biomarker performance in differentiating AD patients from neurological controls. The area under the precision–recall curve (AUPRC) was also computed accounting for unbalanced data. Cutoffs for identification of AD were computed using Youden index to maximize sensitivity and specificity for each CSF biomarker. Confusion matrix was set up in order to calculate sensitivity, specificity, and accuracy at established cutoffs.

Principal component analysis (PCA) was performed on the whole cohort and in the amyloid and tau positive (A + T +) sub-group to explore the pattern of association between the different biomarkers. Outlier values, defined by a value > mean ± 3SD, were excluded for each biomarker before analysis. The suitability of the dataset was evaluated by the Kaiser–Meyer–Olkin Measure of Sampling Adequacy test and Bartlett’s Test of Sphericity. The number of components was determined by the number of eigenvalues greater than one. Variables with a loading factor > 0.4 or < − 0.4 were regarded as representative of the component. Each component was interpreted according to the current understanding of the physiopathology underlying each biomarker in AD. Component scores obtained were compared between groups using linear regression adjusted on age and sex. A two-sided *p* value < 0.05 was considered statistically significant.

## Results

### Gal-3 levels are elevated in the neocortex and hippocampus of post-mortem samples from AD patients

**Fig. 1:**
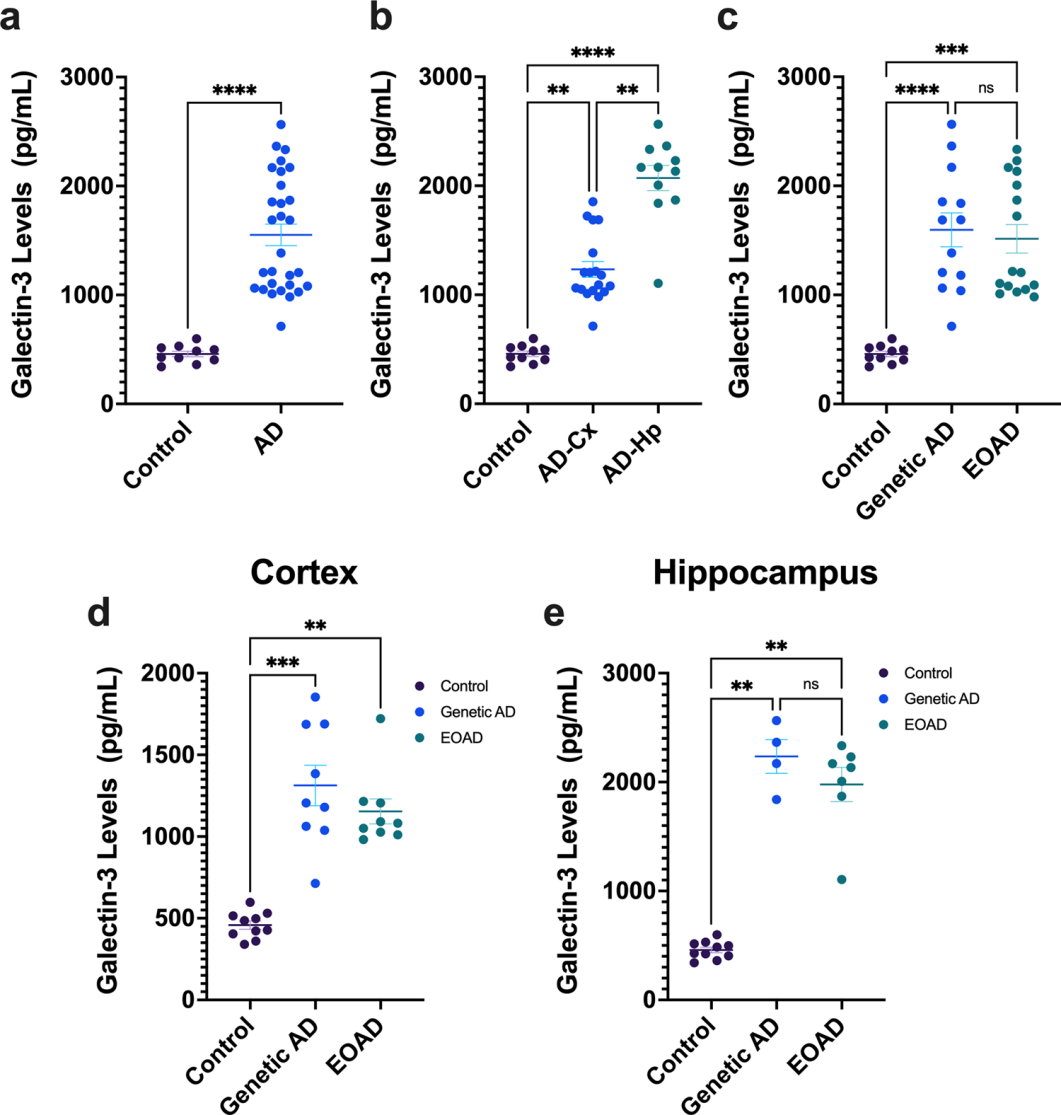
Gal-3 levels are increased in the cortex and the hippocampus in AD patients. First, Gal-3 levels were compared between AD samples versus control samples (**a**). Gal-3 levels measured by ELISA are increased in cortical and hippocampal tissue from AD patients compared to controls (**b**). **c** AD patients were divided into sporadic early-onset AD (EOAD) and genetic AD (PSEN1 mutation) cases. Then, Gal-3 levels were compared between AD groups and controls. **d**, **e** Cortical and hippocampal Gal-3 levels were compared between EOAD and genetic AD groups. Differences were found compared to control samples but not between EOAD and genetic AD groups themselves. Non-parametric t-test **a** and Kruskal–Wallis multiple comparisons (b-e) were performed. Data are shown as mean ± SEM. **p < 0.01; ***p < 0.001. ****p < 0.0001

First, we evaluated the levels of Gal-3 in cortex and hippocampus from AD cases by ELISA (see Supp. Fig. S 1 for demographics). We found Gal-3 levels significantly elevated in AD samples compared to age-matched controls (Fig. 1a). Further analysis showed higher Gal-3 upregulation in the hippocampus compared to the cortex in AD cases (Fig. 1b). Next, to evaluate whether genetic AD cases varied in Gal-3 levels, we divided our cohort into genetic AD cases (*PSEN1* mutation carriers) and sporadic early-onset (EOAD) cases. Our analysis revealed no differences in Gal-3 protein levels between AD groups (genetic AD vs EOAD) (Fig. 1c). However, Gal-3 levels were significantly increased in genetic AD and EOAD samples compared to control (Fig. 1c). Furthermore, we studied regional differences in Gal-3 protein levels between genetic AD and EOAD cases. Therefore, we compared cortical and hippocampal brain tissue but found no differences between the two AD groups (Fig. 1d, e). However, we found that Gal-3 levels in genetic AD and EOAD samples were significantly upregulated in both cortex and hippocampus compared to control cases, suggesting a similar Gal-3-associated immune response in both AD groups. We also confirmed the increase in cortical Gal-3 levels in AD patients compared to controls by western blot (Supp. Fig. S 1g, online resource). Along with Gal-3 levels, we measured Aβ42, t-tau and p-Tau levels. Aβ42 and p-Tau were significantly increased in the frontal cortex and hippocampal samples from AD patients compared to controls (Supp. Fig.  S1a, b, e, f, see online resource). Moreover, Gal-3 levels were not affected by post-mortem time (PMT), age, or sex (Supp. Fig. S2, online resource) in both AD and control samples. Finally, we performed a correlation analysis on Gal-3 levels with Aβ42, t-tau and p-Tau levels. In both control and AD cases, Gal-3 levels in hippocampal or cortical tissue did not correlate with Aβ42, t-tau or p-Tau levels (Supp. Fig. S3, online resource).

### Gal-3-positive microglia is associated with Aβ plaques and tau tangles in AD

**Fig. 2:**
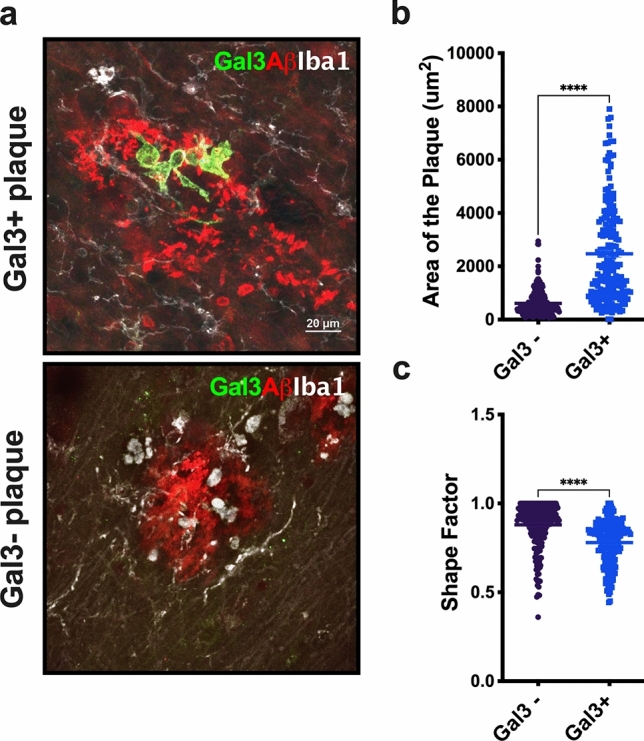
Gal-3-positive microglial cells are associated with larger and more irregularly shaped Aβ plaques. **a** Gal-3-positive microglial cells were associated with larger and more irregularly shaped Aβ plaques (Gal3+ plaques) compared to Gal-3-negative Aβ plaques (Gal3− plaques). **b**, **c** Gal3+ plaques were larger and more irregularly shaped than Gal-3− plaques. Aβ (red), Galectin-3 (green), Iba1 (white). Data are shown as mean ± SEM. Non-parametric t-tests were performed. ****p < 0.0001. (*n* = 3 (HC), *n* = 8 (AD). Gal-3-negative plaques, *n* = 212; Gal-3-positive plaques, *n* = 197)

**Fig. 3:**
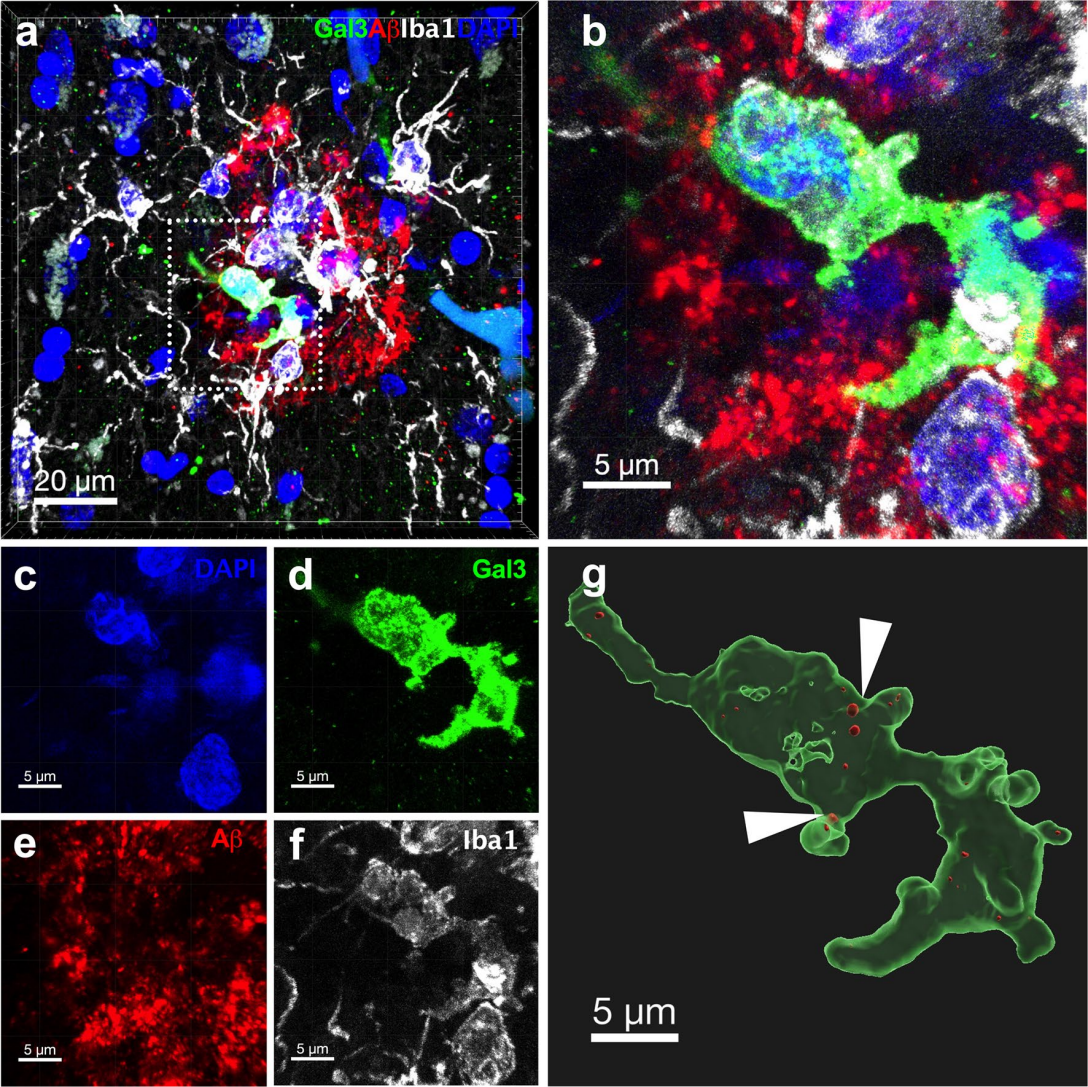
Reactive microglial cells expressing Gal-3 presented Aβ inclusions in human tissue samples. **a**–**f** Gal-3-positive microglial cell associated with Aβ plaques. **g** 3D reconstruction of microglial cells with multiple Aβ inclusions inside. Gal3 (green), Aβ (red), Iba1 (white), DAPI (blue). White arrows are pointing to Aβ inclusions (in red) (*n* = 3 (HC), *n* = 8 (AD)

**Fig. 4:**
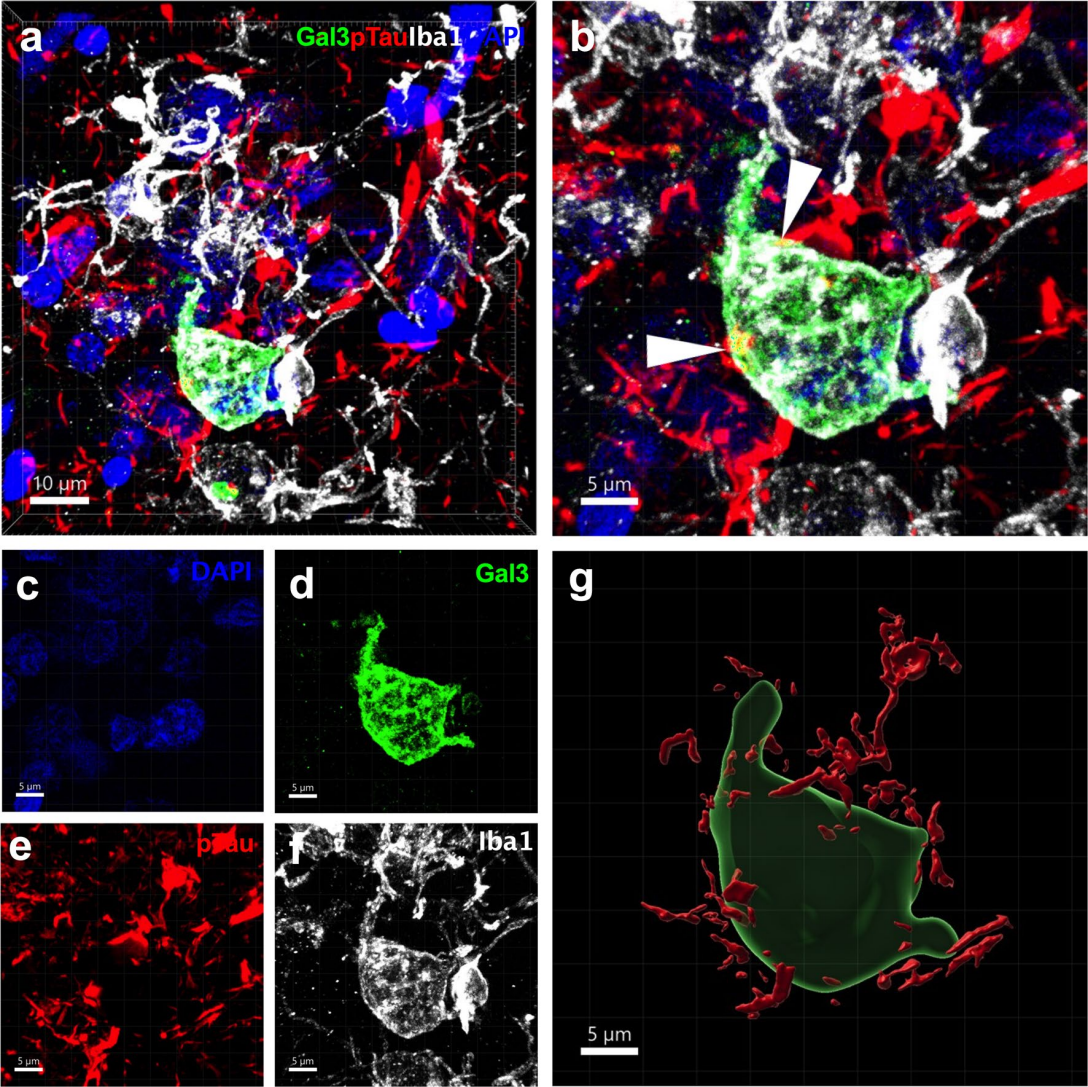
Reactive microglial cells expressing Gal-3 interact with p-Tau in senile plaques from human tissue samples. **a**–**f** Gal-3-positive microglial cell associated with p-Tau plaques. **g** 3D reconstruction of microglial cell with multiple p-Tau interactions. Gal3 (green), p-Tau (red), Iba1 (white) and DAPI (blue). White arrows are pointing p-Tau Gal-3 interactions (in orange). *n* = 3 (HC), *n* = 8 (AD)

We demonstrated a subpopulation of microglia clustered around Aβ plaques identified by high expression of Gal-3[8]. However, we also observed another subset of plaques that were not surrounded by Gal-3-positive microglial cells. Here, we sought to evaluate the main morphological features of Aβ plaques (size and shape) and their association with microglia expressing Gal-3. Plaque size referred to the plaque area as measured in square micrometers based on 6E10 (APP/Aβ) immunolabeling. Plaque shape was defined as a value between 0 and 1. Values closer to 1 indicated a more rounded and regular plaque shape whereas values closer to zero indicated a more irregular plaque shape. To study plaque morphology, we performed triple immunolabeling using the antibodies against Iba1 (microglial marker), 6E10 (APP/Aβ), and Gal-3 on human AD cortical tissue (Fig. 2a). Notably, clear morphological differences were observed between Gal-3-positive and Gal-3-negative plaques. We defined Gal-3-positive and Gal-3-negative plaques as those surrounded by microglial cells expressing or lacking Gal-3, respectively. Gal-3-positive plaques covered a larger area (Fig. 2b) and had a more irregular shape (Fig. 2c) compared to Gal-3-negative plaques. Our analysis revealed that microglial cells expressing Gal-3 were associated with Aβ plaques (Fig. 3a–g), which may indicate plaque phagocytosis by the microglia [13, 23, 60, 62]. Indeed, microglial cells expressing Gal-3 near plaques presented numerous Aβ inclusions (Fig. 3, white arrows). Moreover, we also found Gal-3-positive microglial cells close to p-Tau aggregates in senile plaques (Fig. 4a–g). Notably, 3D modeling revealed p-Tau interacting with Gal-3-positive microglial cells in what is likely a senile plaque containing p-Tau-aggregates (Fig. 4b, white arrows).

### CSF Gal-3 levels are increased in AD and are associated with neuroinflammatory alterations

**Fig. 5:**
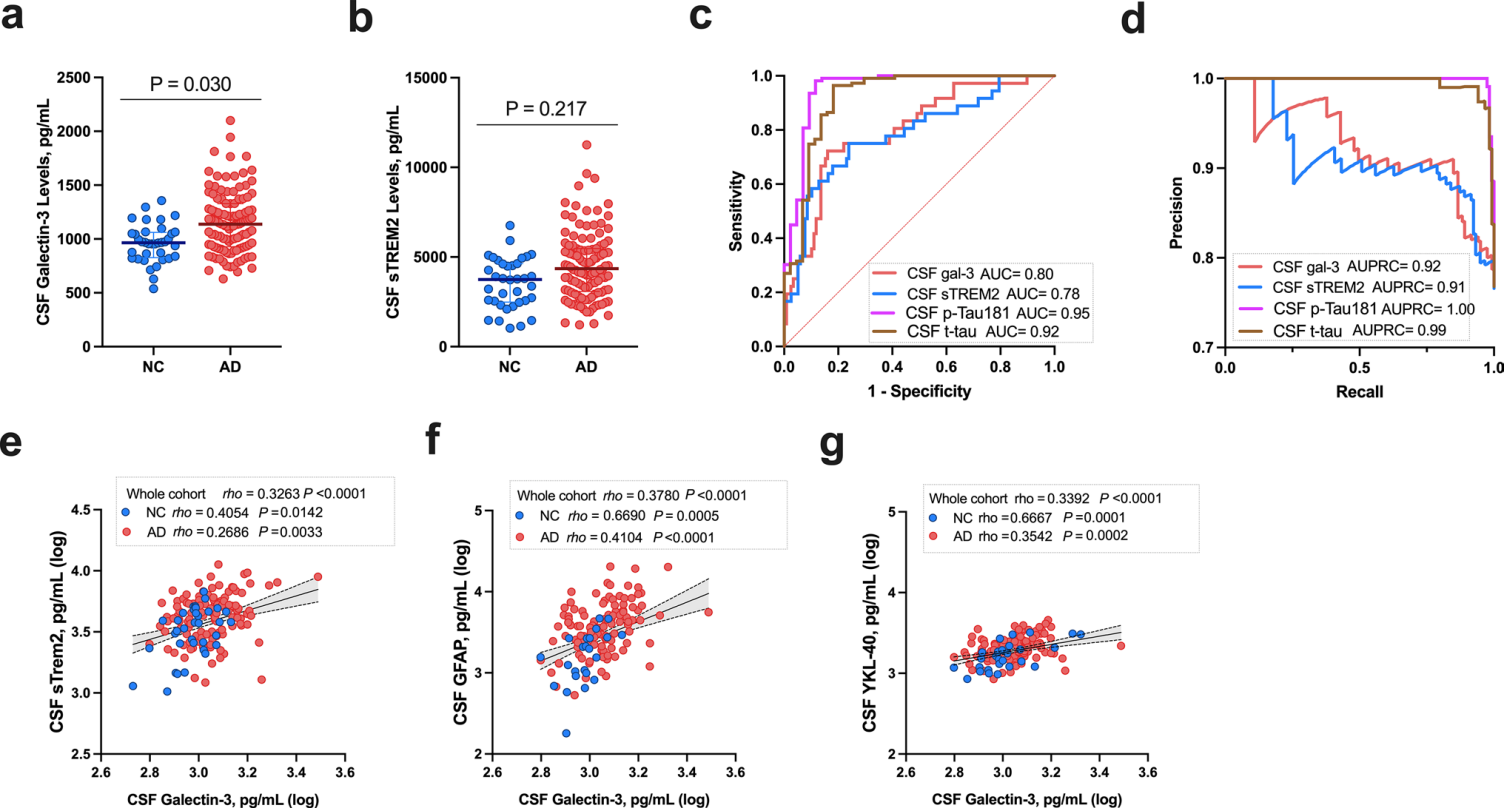
CSF Gal-3 levels are increased in AD patients and correlate with other CSF neuroinflammatory biomarkers. **a** CSF Gal-3 levels were measured by ELISA in control subjects and AD patients. Gal-3 levels were significantly elevated in AD patients compared to controls (*P = 0.030) after adjustment on age, sex and ApoE4 carriership. **b** CSF sTREM-2 levels were measured in our cohort. No difference was found between the AD and control groups in our analysis adjusted for age, sex and ApoE4 carriership (*P* = 0.217). **c**, **d** Analysis of ROC curves revealed moderate performance of CSF Gal-3 and sTREM-2 levels for differentiating AD patients from neurological controls (**c** Gal-3 AUROC = 0.80 [95% CI = 0.72–0.88], sTREM-2 AUROC = 0.78 [95% CI = 0.69–0.88]; **d** Gal-3 AUPRC = 0.92, sTREM-2 AUPRC = 0.91). For comparison, CSF markers p-tau and t-tau demonstrated high discriminating performance between AD and controls (c: p-tau AUROC = 0.95 [95% CI = 0.91–1.00], t-tau AUROC = 0.92 [95% CI = 0.86–0.98]; 5d: p-tau AUPRC = 1.00, t-tau AUPRC = 0.99). **e**,** f**,** g** The relationships between CSF Gal-3 and other CSF neuroinflammatory biomarkers—sTREM-2, GFAP and YKL-40—were studied using Spearman’s rank correlation in the whole cohort as well as in AD and neurological control (NC) subgroups

We then analyzed CSF samples from a cohort including 119 patients with AD and 36 neurological controls (NC). The cohorts and CSF biomarkers levels are described in Table 1. AD patients were significantly older than NC (*P* < 0.001) and displayed higher ApoE4 carriership *(P* < 0.001). First, we focused our investigation on determining the relationship between CSF Gal-3 and AD characteristics. We found that CSF Gal-3 levels correlated positively with age (rho = 0.402, *P* < 0.001). However, Gal-3 did not associate significantly with sex (*P* = 0.079) or *APOE-*ε4 carrier status (*P* = 0.432). Overall, AD patients displayed higher CSF Gal-3 levels compared to NC (1168.8 pg/mL versus 960.5 pg/mL, *P* = 0.030 adjusted for age, sex, and ApoE4 carriership Fig. 5a). The CSF profile in relation to the AD continuum showed that Gal-3 levels did not differ between A + T−N−, A + T + N− and A + T + N + groups (*P* = 0.440).

We next analyzed neuroinflammatory-related markers in CSF and considered their relationship with CSF Gal-3. We found that sTREM-2 levels did not significantly differ between NC and AD groups (*P* = 0.217, Fig. 5b). To distinguish AD patients from NC, CSF Gal-3 and sTREM-2 levels were moderately good predictors (AUROC = 0.80 and AUROC = 0.78, respectively, Fig. 5c). However, their performance remained lower than CSF p-Tau and t-tau, the gold standard for diagnosis (respectively, AUROC = 0.95 and AUROC = 0.92). Detailed comparison of sensitivity, specificity and accuracy at optimal cutoff designed through ROC analysis are available for each biomarker in Supp. Table 2 (online resources) and Supp. Fig. S4 (online resources).

Relating these two factors, we observed that Gal-3 weakly correlated with sTREM-2 (rho = 0.326, *P* < 0.0001, Fig. 5e). This correlation held when looking specifically in AD patients and the NC group (rho = 0.269, *P* = 0.0033 and rho = 0.405, *P* = 0.0142, respectively). In addition, CSF Gal-3 weakly correlated with GFAP (rho = 0.378, *P* < 0.0001, Fig. 5f) and YKL-40 (rho = 0.339, *P* < 0.0001, Fig. 5g) when looking at the whole cohort. These correlations were sustained for both markers in the AD patient group (GFAP: AD, rho = 0. 410, *P* < 0.0001; YKL-40: AD, rho = 0.354, *P* = 0.0002) but not in the NC group. We used the CSF/plasma albumin quotient to indirectly study the relationship between Gal-3 and brain blood barrier integrity. CSF Gal-3 levels correlated very moderately with the CSF/plasma albumin quotient in the whole cohort (rho = 0.255, *P* = 0.0043) and in the AD group (rho = 0.263, *P* = 0.0067). Regarding the other CSF biomarkers, only CSF GFAP was associated with the CSF/plasma albumin quotient in the AD group (Supp. Table S3, online resources).

### Gal-3 levels are associated with tau and synaptic marker levels in CSF in AD

**Fig. 6:**
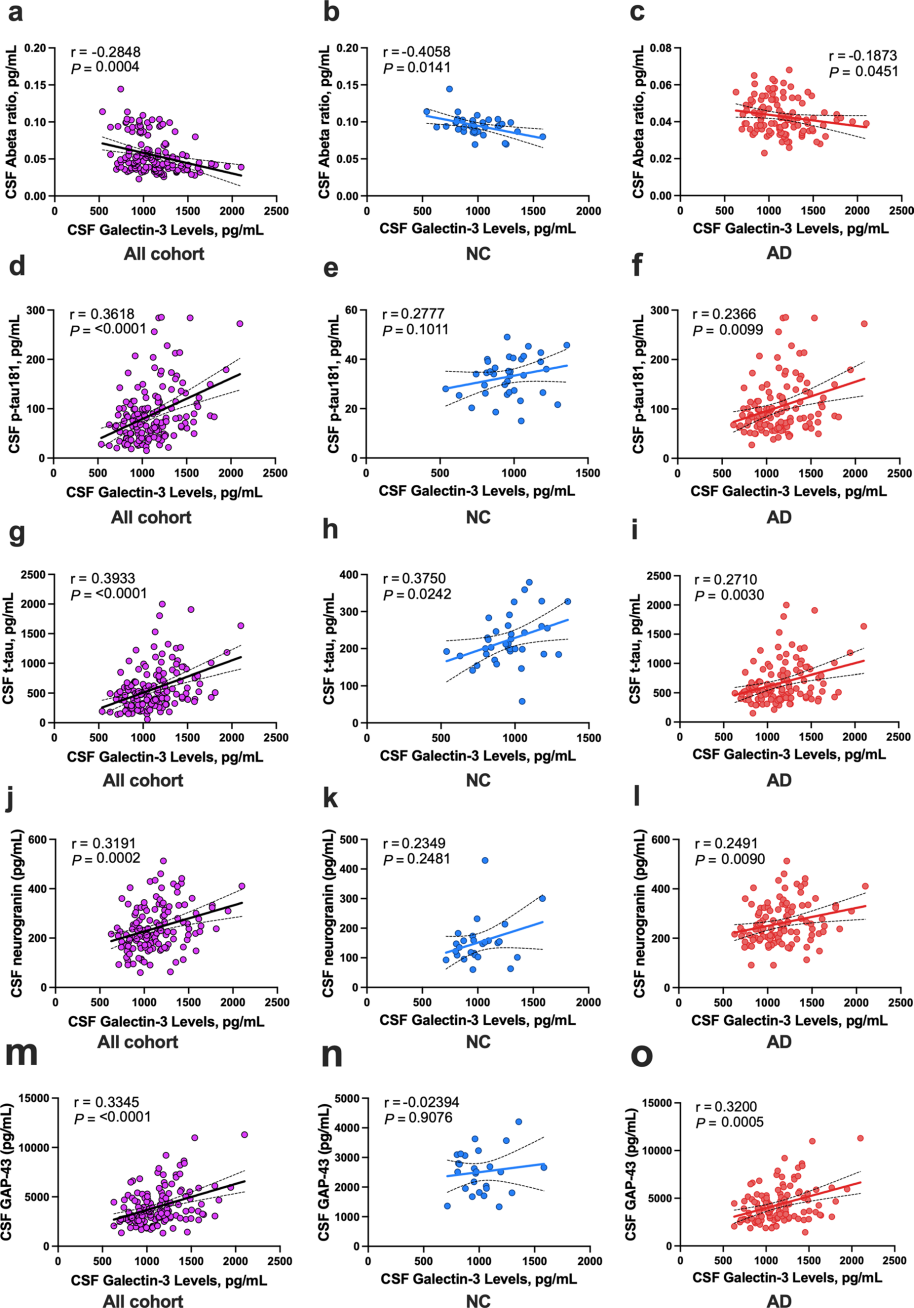
CSF Gal-3 levels correlate with CSF tau and synaptic markers. **a**–**o** Scatter plots depicting the association between CSF Gal-3 levels with other CSF AD and synaptic markers in the whole cohort and in the sub-groups (neurological controls [NC] *n* = 36 and AD *n* = 119). **a**–**c** CSF Aβ ratio weakly correlated with CSF Gal-3 in the whole cohort and sub-groups. **d**–**i** CSF Gal-3 significantly correlated with CSF p-tau181 and t-tau in the whole cohort and some sub-groups. **j**–**o** CSF Gal-3 levels correlated with CSF synaptic markers neurogranin **j**–**l** and GAP-43 **m**–**o** in the whole cohort and in the AD patient sub-group. Associations were assessed using Spearman’s rank correlation. Solid line indicates regression line, and dotted lines border the 95% confidence interval

We next looked at the relationship between CSF Gal-3 levels and other CSF biomarkers of AD. Gal-3 levels were negatively correlated with Aβ42/Aβ40 ratio (rho = − 0.285, *P* = 0.0004, Fig. 6a) in the whole cohort as well as in the NC (rho = − 0.406, *P* = 0.0141) and AD patient (rho = − 0.187, *P* = 0.0451) sub-groups (Fig. 6b, c). In contrast, Gal-3 levels were positively correlated with p-Tau181 in the whole cohort (rho = 0.362, *P* < 0.0001), but of the sub-groups, only the AD cohort maintained a statistically significant correlation (rho = 0.237, *P* = 0.0099, Fig. 6d–f). Similarly, CSF Gal-3 positively correlated with t-tau (rho = 0.393, *P* < 0.0001) in the whole cohort and separately in the AD cohort (rho = 0.271, *P* = 0.003) and the NC group (rho = 0.375, *P* = 0.024) (Fig. 6g–i). Looking at the relationship between CSF Gal-3 and CSF synaptic biomarkers, we found that Gal-3 positively correlated with neurogranin and GAP-43 when including the whole cohort (rho = 0.319, *P* = 0.0002 and rho = 0.334, *P* < 0.0001, respectively, Fig. 6j, m). This statistically significant correlation was reflected in the AD group for both neurogranin (rho = 0.249, *P* = 0.0090, Fig. 6l) and GAP-43 (rho = 0.320, *P* = 0.0005, Fig. 6o) but not in the NC group.

### CSF Gal-3 levels correlate with cognitive decline in AD

We studied the association between CSF Gal-3 levels and general cognition via Mini-Mental State Exam (MMSE) scores (Supp. Fig. S5a, online resource). Gal-3 levels were associated with MMSE scores in AD patients, independently of sex, age and level of education (*β* = 0.176, 95% CI = 0.010 to 0.341, *P* = 0.0217). However, Gal-3 levels were not associated with MMSE scores after adjusting for age, sex and level of education when looking at the whole cohort (*β* = − 0.042, 95% CI = − 0.202–0.118, *P* = 0.605) nor in the NC cohort (*β* = 0.010, 95% CI = − 0.371–0.391, *P* = 0.957). We performed regression analysis to study the relationship between CSF Gal-3 levels with MMSE scores in the AD cohort (Supp. Fig. S5b, online resource). A significant quadratic function was found for the relationship between CSF Gal-3 levels and MMSE scores in AD patients (*P* = 0.039). This could be depicted as an inverse U-shaped curve showing that higher levels of CSF Gal-3 were associated with intermediate MMSE scores. However, lower Gal-3 levels were associated with the highest and lowest MMSE scores in AD patients.

### CSF Gal-3 clusters with neuroinflammatory CSF biomarkers in PCA analysis

**Fig. 7:**
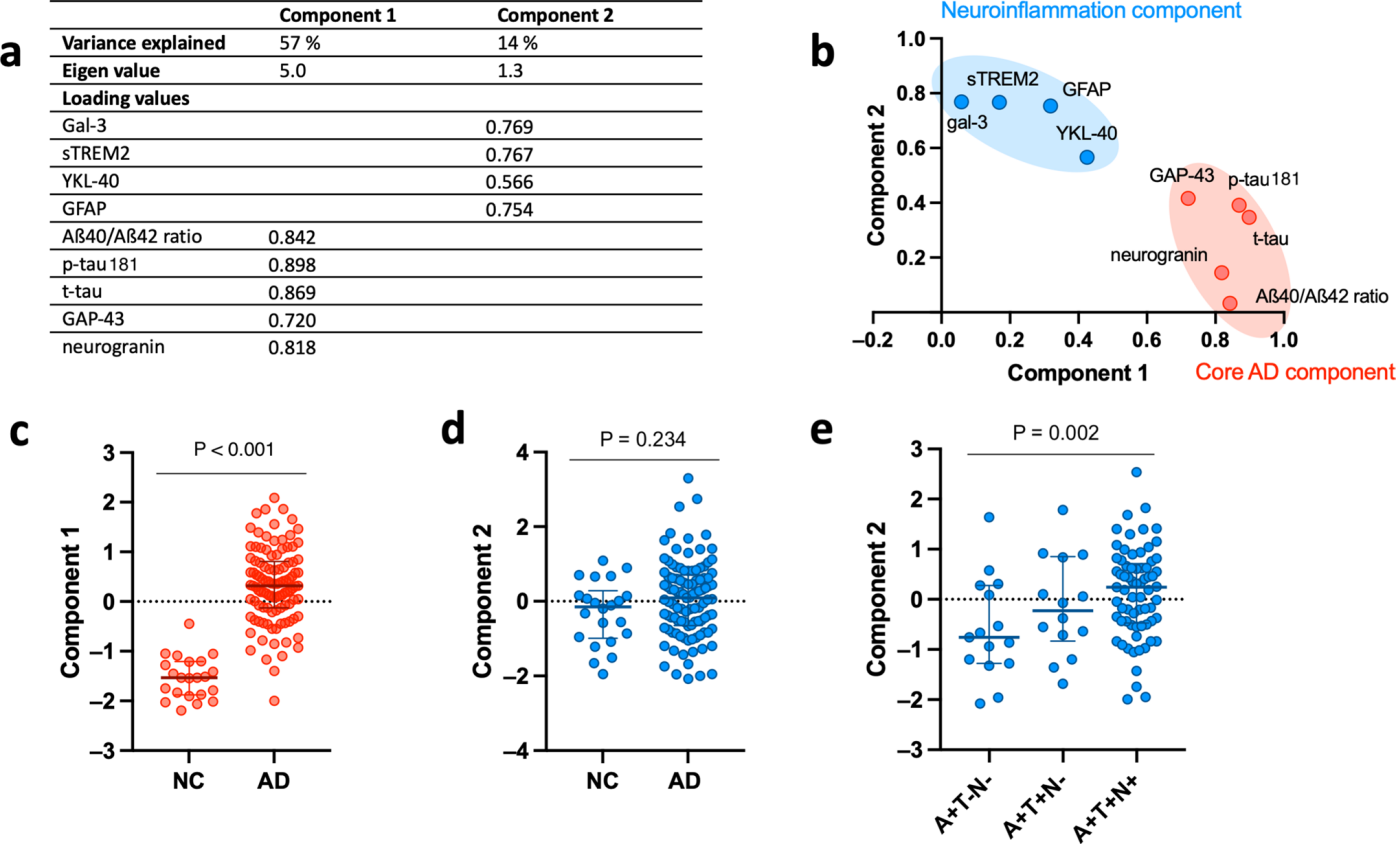
CSF Gal-3 clusters with a neuroinflammatory component in principal component analysis. **a**, **b** Principal component analysis (PCA) in the whole cohort revealed clustering of the CSF biomarkers in two principal components (**a** loading values of each CSF biomarker, eigenvalues, and variance explained for each component identified; **b**: scree-plot in Varimax rotation). Component 1 is associated with CSF core AD biomarkers (Aß ratio, p-Tau181, and t-tau) and CSF synaptic markers (neurogranin, GAP-43). Component 2 included the CSF neuroinflammation markers (Gal-3, sTREM-2, GFAP, and YKL-40). Component 1 and Component 2 accounted for 57% and 14% of the variance. **c**–**e** Identified components were compared between groups using one-way ANCOVA-adjusted on age and sex followed by post hoc Least square test, adjusted with Bonferroni for multiple comparisons. **c** Core AD Component 1 was significantly higher in AD patients than in all other groups (****P < 0.0001 versus all other groups). **d** Neuroinflammation Component 2 did not differ between the groups. **e** Focusing on the patients on the AD continuum, neuroinflammatory Component 2 was significantly higher in patients with a [A + T + N +] CSF profile compared to patients with an [A + T−N−] profile (*P* = 0.002)

Lastly, we performed PCA to investigate the relationship between the different biomarkers. We identified 2 principal components that explained 71% of the total variance in the dataset (Fig. 7a, b). Component 1 (PC1) accounted for 57% of the variance and was associated with core AD biomarkers: Aβ40/Aβ42, p-Tau181, t-tau, and the synaptic markers neurogranin and GAP-43. Component 2 (PC2) captured 14% of the variance and was associated with neuroinflammatory markers Gal-3, sTREM-2, YKL-40 and GFAP. Notably, in the neuroinflammatory component PC2, the marker with the highest weight was Gal-3. In the core AD component PC1, p-Tau181 was the leading marker. After adjusting for age and sex, PC1 was significantly increased in the AD group compared to NC (*P* < 0.0001, Fig. 7c). Neuroinflammation PC2 did not differ between groups after adjusting for multiple comparisons (Fig. 7d). However, of all AD patients, PC2 was significantly higher in the A + T + N + group compared to the A + T−N− group (*P* = 0.002, Fig. 7e). PCA analysis was further performed on the A + T + AD patient group, and the same two components, core AD PC1 and neuroinflammation PC2 could be detected (Supp. Fig. S6, online resources). Interestingly, in A + T + subjects, PC1 and PC2 had a quadratic relationship, wherein PC2 had a U-shaped form following increasing levels of core AD PC1 (*P* = 0.031) (Supp. Fig. S6c, online resource).

## Discussion

In the present study, we assessed the microglial activity marker Gal-3 in clinically diagnosed and neuropathologically confirmed AD patients and analyzed the relationship between CSF levels of Gal-3 and AD markers and characteristics in a clinical cohort. Our data showed an upregulation of Gal-3 in cortical and hippocampal tissue from sporadic EOAD and genetic AD cases compared to controls, further highlighting Gal-3-specific microglial activation in AD brain related to Aβ plaque deposits. Gal-3 levels were not associated with age or post-mortem time delay. A detailed analysis demonstrated that Gal-3-positive microglia associated more frequently with Aβ plaques that were large and irregular and associated with neurons with p-Tau inclusions in human brain tissue samples. Complementing this, we measured CSF Gal-3 levels and other CSF AD biomarkers in AD patients. Like in tissue, we found higher CSF Gal-3 levels in AD patients compared to control subjects. CSF Gal-3 levels correlated with markers of neuronal degeneration (tau and p-Tau181), synaptic dysfunction (neurogranin and GAP-43), and to an even greater extent, inflammatory markers (GFAP, YKL-40 and sTREM-2). Lastly, we performed PCA and found that neuroinflammatory markers cluster together separately from the traditional hallmarks of the pathology. With this study, we expanded on our work demonstrating the role of Gal-3 on the detrimental inflammatory response in AD and show, in human CSF, that Gal-3 is associated with core biomarkers of AD and with neuroinflammatory markers.

In AD, Gal-3 is preferentially expressed by activated microglia and is released into the extracellular space. Because of this, Gal-3 can be detected in CSF and serum [3, 8, 10, 55, 59]. We sought to explore the potential of Gal-3 as an AD biomarker by analyzing the relationship of Gal-3 with markers of pathology progression and cognitive decline in CSF. Compared to controls, we have observed that AD patients have significantly higher Gal-3 levels in the brain, both in this study and our previous one [10]. Moreover, Gal-3 levels were higher in hippocampus compared to frontal cortex. This may be related to the larger size of Aβ deposits in hippocampus and, therefore, more robust microglial activation. Indeed, in our previous study, we demonstrated Gal-3-dependent microglial activation that took place only in the vicinity of Aβ plaques [8]. An increase in Gal-3 makes sense given its source and the findings demonstrating an increase in microglial activity in AD [58]. In our study, the amyloid plaque morphology associated with Gal-3-positive plaques resembled the recently named coarse-grained plaques [7]. Notably, coarse-grained plaques are associated with intense neuroinflammation (CD68 and MHCII positive), ApoE4, and vascular pathology [16].

Interestingly, Gal-3+ microglia contained a notable number of Aβ inclusions, highlighting their phagocytic capacity associated with the pathology [62]. The receptors involved in microglial cells phagocytosis of Aβ plaques are not fully understood. Recently, the importance of two specific TAM receptors, Axl and MerTK, has been highlighted in the detection and engulfment of Aβ plaques [23]. This was demonstrated in an APP mouse model lacking Axl and Mer which had impaired detection and engulfment of Aβ plaques. Importantly, Gal-3 is a ligand for MerTk and thereby stimulates phagocytosis. Therefore, the Aβ inclusion observed inside microglial cells expressing Gal-3 may be partially mediated by Axl, MerTK and Gal-3 interaction [12]. We also describe Gal-3+ microglia close to neurons with p-Tau aggregates in human senile plaques, suggesting a relation between microglial activation and p-Tau aggregates. Recently, Pascoal et al. demonstrated the joint propagation of tau-pathology and microglial activation in AD [40]. Indeed, this study revealed that tau propagation is more dependent on microglial networks than tau network circuits [40]. A key regulator of tau propagation is Low-Density Lipoprotein 1 receptor (LRP1), which has been related to tau uptake and spreading [43] and also to the microglial inflammatory response [57]. Notably, galectin-3 binding studies performed on mesenchymal retinal cells have demonstrated a high binding affinity between galectin-3 and LRP1 [38]. In regards to tau-dependent microglial activation, Jin et al. discovered a role of polyglutamine binding protein 1 (PQBP1) in sensing extracellular tau and an associated microglial pro-inflammatory response. This microglial inflammatory response is dependent on NFκB-dependent transcription of inflammatory genes, leading to brain inflammation [27]. Despite all the progress, little is known about the mechanisms involving extracellular tau-dependent microglial activation and further experiments are needed to address this question.

Traditionally, Aβ, p-Tau and tau are the main diagnostic biomarkers for AD used in clinical practice [16]. However, less is known whether these biomarkers are associated with other key neuropathological features, including neuroinflammation, vascular impairment and blood–brain barrier disruption. Our data looking at CSF consistently underline the relation of Gal-3 with other key CSF biomarkers in AD progression. Higher Gal-3 levels correlated with tau and p-Tau181 levels, two indicators of pathology progression in AD. Indeed, microglial activation progresses along with tau deposition across the different Braak stages, indicating the cooperation of both phenomena [40]. Indeed, the combination of neuroinflammatory microglial activity and tau deposition measured by PET predicts cognitive decline in AD [35]. The latest is consistent with the pathological presentation of microglial cells expressing Gal-3.

Together with tau-pathology biomarkers, Gal-3 levels strongly correlated with CSF markers of synaptic dysfunction, GAP-43, and neurogranin. Synaptic failure is an early process of AD, and synapse loss is a neuropathological hallmark in connection with cognitive decline. Physiologically, microglia have been implicated in maintaining functional synaptic connections and plasticity [36]. Synaptic dysfunction is an early feature in AD, and recent studies suggest that microglia-mediated synapse removal could be a contributing factor [22]. In AD, microglia constitute a cellular mediator of synapse loss through phagocytosis or release of synaptotoxic factors [9, 22, 36, 45]. In our work, CSF Gal-3 correlated with pre-synaptic protein GAP-43, which displays a significant increase in AD and correlates with the magnitude of neurofibrillary tangles and Aβ plaques [44]. CSF Gal-3 also correlates with levels of neurogranin, a post-synaptic protein consistently increased in CSF in AD [41]. Neurogranin levels are positively correlated with increased neuritic plaques [41].

In our study, Gal-3 levels weakly correlated with CSF neuroinflammatory markers YKL-40, GFAP and sTREM-2. Notably, in our cohort, Gal-3 demonstrated better sensitivity and specificity than sTREM-2 to differentiate AD patients from neurological controls. In AD, YKL-40 has been found to be elevated and associated with astrocyte reactivity [31, 34, 42]. Like YKL-40, GFAP has been linked to astrocyte activity and found to be consistently elevated in CSF and serum of patients in preclinical and symptomatic AD stages [6, 24, 39]. The correlations with the neuroinflammatory markers were weaker than neuronal or synaptic markers, suggesting that Gal-3 monitors complementary inflammatory processes differently than those monitored by GFAP, sTREM-2 or YKL-40. sTREM-2 is considered a microglial marker in AD [51]. In our study, sTREM-2 was slightly but not significantly upregulated in AD samples compared to cognitively normal samples. Other studies have pointed out significantly elevated levels of sTREM-2 in CSF from AD samples compared to control. It is important to note that these studies were longitudinal studies on genetic-case cohorts (DIAN) or studies focused on the early stages of AD pathology [17, 18, 50, 51]. In this translational work, we studied CSF Gal-3 in a discovery cohort to explore how it could translate as a CSF biomarker of the microglial response. Brain expression of TREM-2 has also been linked to disease-associated microglial phenotype and plaque growth dynamics [29, 32, 54, 61]. The lack of TREM-2 has been linked to a more diffuse amyloid plaque growth, leading to increased formation of dystrophic neurites [61]. In clinical studies, higher levels of sTREM-2 have been linked to reducing cognitive decline and clinical decline [18] and lower ratios of amyloid-beta [17], which might be linked to its role in plaque formation as described by Yuan et al. [61]

Our PCA analysis displayed a U-shape relation between PC1 (Core AD biomarkers) and PC2 (Neuroinflammatory biomarkers) that might indicate two different stages of inflammatory response throughout the pathology. Indeed, neuroinflammation and microglia have been shown to be increasingly important in AD progression. However, whether microglia and the inflammatory process can and should be judged as beneficial or harmful is often debated. These debates have been fueled by the discovery of specific microglial phenotypes, namely disease-associated microglia (DAM) and neurodegenerative microglia (MGnD) [29, 30]. The DAM phenotype involves TREM-2 signaling and critical genes, such as *Axl*, *cst7*, *lpl or cd9*. On the other hand, the MGnD phenotype, discovered by Krasemann et al. [30], depends on TREM-2-ApoE signaling, shares similarities with the DAM phenotype, and involves key genes such as *Gal-3*, *Clec7a*, *Itgax and Spp1*. Therefore, we can distinguish two stages linked to pathology progression: a primary stage when the neuroinflammatory response occurs in patients with lower levels of amyloid-beta and tau relying on TREM-2 expression, and a second stage when a patient presents with a more advanced stage of the pathology with Gal-3 playing a prominent role. Indeed, Gal-3-positive microglia lead to a pro-inflammatory microglial phenotype that might be unrelated to the TREM-2-dependent phenotype (DAM) but closer to the ApoE-dependent MGnD phenotype [30]. Therefore, TREM-2-dependent microglial activation and Gal-3-dependent microglial activation might represent a sequential process initiated by TREM-2 to counteract the progression of the pathology followed by a Gal-3-dependent secondary response leading to a pro-inflammatory and more detrimental microglial phenotype. Nevertheless, some of the genes involved in each phenotype are shared, and the different phenotypes may co-exist. Understanding the precise role of Gal-3 in these phenotypes as well as in AD pathology is important, especially so if Gal-3 is considered as a potential neuroinflammatory biomarker and a druggable target for AD. Indeed, clinical trials are underway targeting Gal-3 with an antibody-based treatment (Clinicaltrials.gov (NCT05156827)).

Lastly, the cognitive evaluation revealed a quadratic relation between Gal-3 levels and MMSE score. This quadratic relation might reflect the evolution of microglial activation over different stages of the pathology [33, 37]. This kinetic could indicate the progression of microglial activity over pathology progression: a primary phase wherein Gal-3 levels increase and correspond with MMSE scores in all the way into the intermediate stage of AD pathology. However, Gal-3 levels are reduced in the second phase and correspond to the lowest MMSE scores. This may indicate that in the latest stage of AD pathology, microglial cells become dystrophic [47, 49] with impaired functionality, activation capacity and Gal-3 production and release.

We note several limitations in our work. Regarding the CSF study, we recognize that due to a limited number of subjects, we lacked the power needed to explore subtler differences between groups with certain biomarkers, notably sTREM-2. Moreover, there was a significant age gap between AD patients and NC, which could constitute a confounding factor even though the analysis was adjusted for the age difference. Finally, due to the cross-sectional nature of this study, we were unable to depict CSF Gal-3 changes within individuals as they progressed through the AD continuum.

Finding new biomarkers to complement current methods is needed not only for early diagnosis, but also for improving the design of clinical trials and monitoring the effectiveness of disease-modifying therapies. Microglial activation, Aβ plaques and tau aggregation are key in neuronal dysfunction. We demonstrate that Gal-3 is strongly associated with the core biomarkers in AD pathology, and, like others, that Gal-3 is a key mediator of the microglial pro-inflammatory phenotype in AD [28, 30].

## Supplementary Information

Below is the link to the electronic supplementary material.

Supplementary file1. Fig. 1. Aβ42 and p-Tau levels were upregulated in cortical and hippocampal samples from AD patients. a-b Aβ42 levels were significantly upregulated in cortical (AD-Cx) and hippocampal (AD-Hc) samples from AD patients compared to control samples. c-d) Total Tau levels were not significantly different between AD patients and controls in the cortex (AD-Cx) or hippocampus (AD-Hc). e–f) Phospho-Tau (p-Tau) levels were significantly upregulated in cortical (AD-Cx) and hippocampal (AD-Hc) samples from AD patients compared to control samples. g) Gal-3 levels from cortical samples from AD patients and controls were analyzed by western blot. Our data confirmed a clear up-regulation of Gal-3 in AD patients). Data are shown as Mean ± SEM. A parametric t-test was applied. *p < 0,05; **p < 0.01; ***p < 0.001; ****p < 0.0001. Fig. 2. Gal-3 levels are not affected by age, sex or post-mortem time in brain tissue from AD patients. a-d) Correlation analysis in control and AD brain samples was performed to evaluate whether age, sex, or post-mortem time induces an up-regulation of Gal-3. No significant correlation was found for any of the parameters evaluated. Simple regression analysis was performed in each case. Fig. 3. Aβ42 and tau levels do not correlate with Gal-3 levels in brain tissue of AD patients. a-c) Correlation analysis in cortex and hippocampus from controls and AD patients was performed. No significant differences were found in the analyses performed. c) A positive trend between Gal-3 and Aβ42 was observed in hippocampal samples from AD patients. Simple regression analysis was performed in each case. d-f) Correlation analysis in cortex and hippocampus from controls and AD patients were performed. No significant differences were found in any analysis performed. f) Positive trends between Gal-3 and Total Tau (Tau) and Gal-3 and p-Tau were found in hippocampal samples from AD patients. Simple regression analysis was performed in each case. Fig. 4. Confusions matrix for each CSF biomarker for identification of AD. Confusion matrix for a, CSF Gal-3; b, CSF p-tau; c, STREM2; d, CSF tau for identification of AD, depicting numbers of TN, FN, TP and FP. AD, Alzheimer's disease; CSF, cerebropsinal fluid; FP, false positive; FN, false negative; NC, neurological controls; TP, true positive; TN, true negative. Fig. 5. CSF Gal-3 levels are associated with cognitive impairment in AD. a) The relationship between MMSE scores and CSF Gal-3 levels was explored through linear regression adjusted for age, sex and level of education in the whole cohort and the different subgroups. Before analysis, MMSE scores were transformed into the square root of the number of errors to improve data distribution. A significant inverse correlation was found between MMSE scores and CSF Gal-3 levels in the AD group. b) In patients with AD, CSF Gal-3 levels fit into quadratic relationship with MMSE scores. Linear and quadratic regression models were assessed with CSF Gal-3 (ie, linear) and their squared term (ie, quadratic) as independent variables, and MMSE as the dependent variable. As both the linear and quadratic models were significant based on the F statistic, the Akaike information criterion (AIC) was used to determine which model better fit the data, with a smaller AIC indicating a better fit. A difference of > 2 in the AIC was considered a significant difference between models. Quadratic regression provided a significantly better fit than linear regression (difference in Akaike information criterion between quadratic and linear regression, ∂AIc = 2.111). Fig. 6. Neuroinflammatory component PC2 displays a U-shaped relationship with the core AD component in A + T + subjects. a-b) PCA analysis of AD patients with an [A + T +] CSF profile clustered the CSF biomarkers in the same two PCs identified in the whole cohort analysis a.) loading values and variance explained; b: scree-plot). Component 1 associated core AD biomarkers (Aß ratio, p-Tau181, and t-tau) and synaptic markers (neurogranin, GAP-43). Component 2 included the neuroinflammation markers (Gal-3, sTREM-2, GFAP, and YKL-40). PC1 and PC2 accounted for 49% and 13% of the variance. c) Neuroinflammatory component PC2 displayed a significant U-shaped relationship with core AD component PC1. Quadratic regression was performed and provided a significantly better fit than linear regression (difference in Akaike information criterion between quadratic regression and linear regression, ∂AIc = 12.08). (PDF 2500 KB)

Supplementary file2 (PDF 119 KB)
