# Supplementary material for: Galectin-3 is elevated in CSF and is associated with Aβ deposits and tau aggregates in brain tissue in Alzheimer’s disease
Source: Acta Neuropathol. 2022 Jul 27;144(5):843–59. doi: 10.1007/s00401-022-02469-6 (PMC9547798; doi:10.1007/s00401-022-02469-6)

Supp. Figure 1

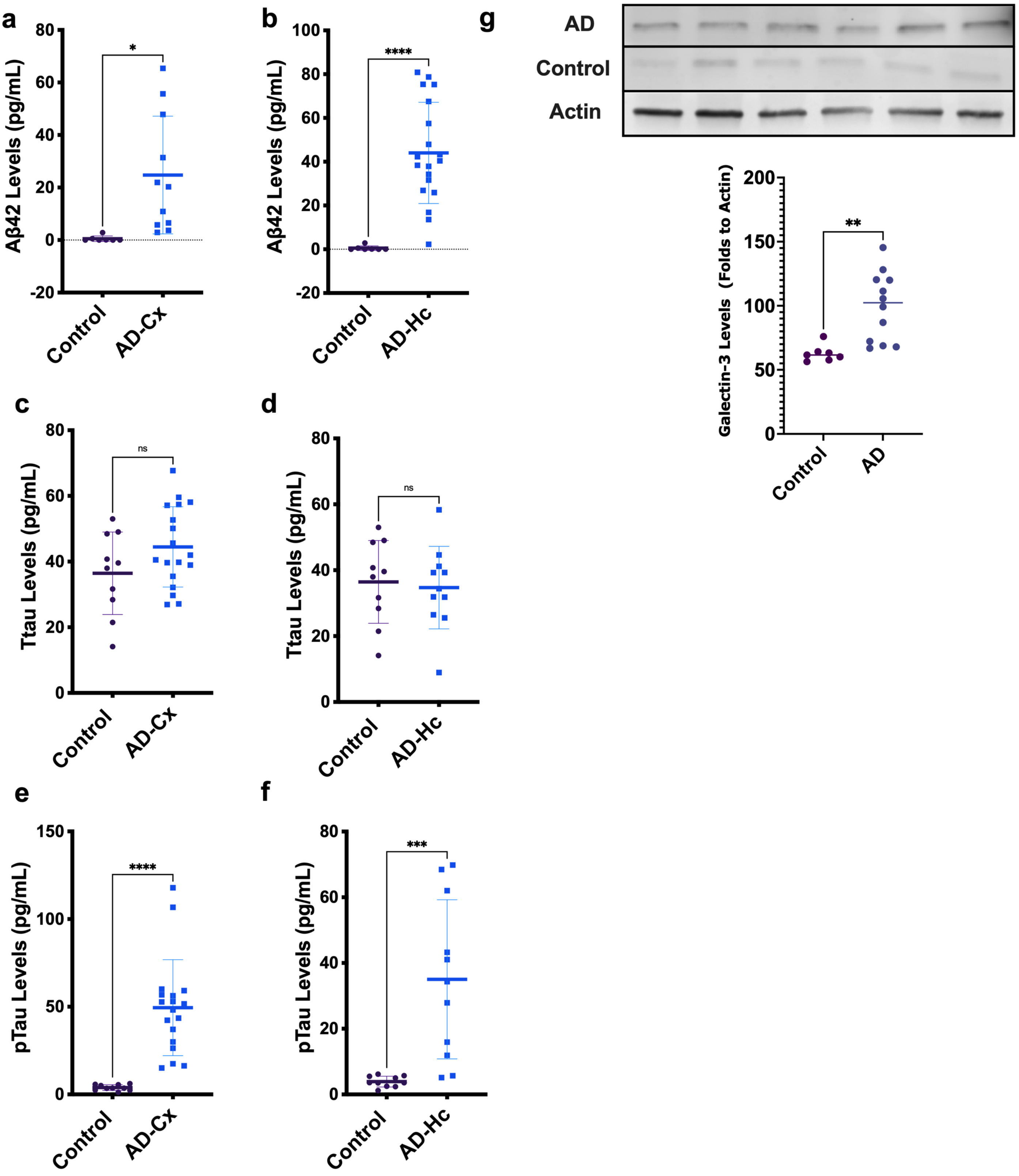

Supp. Figure 2

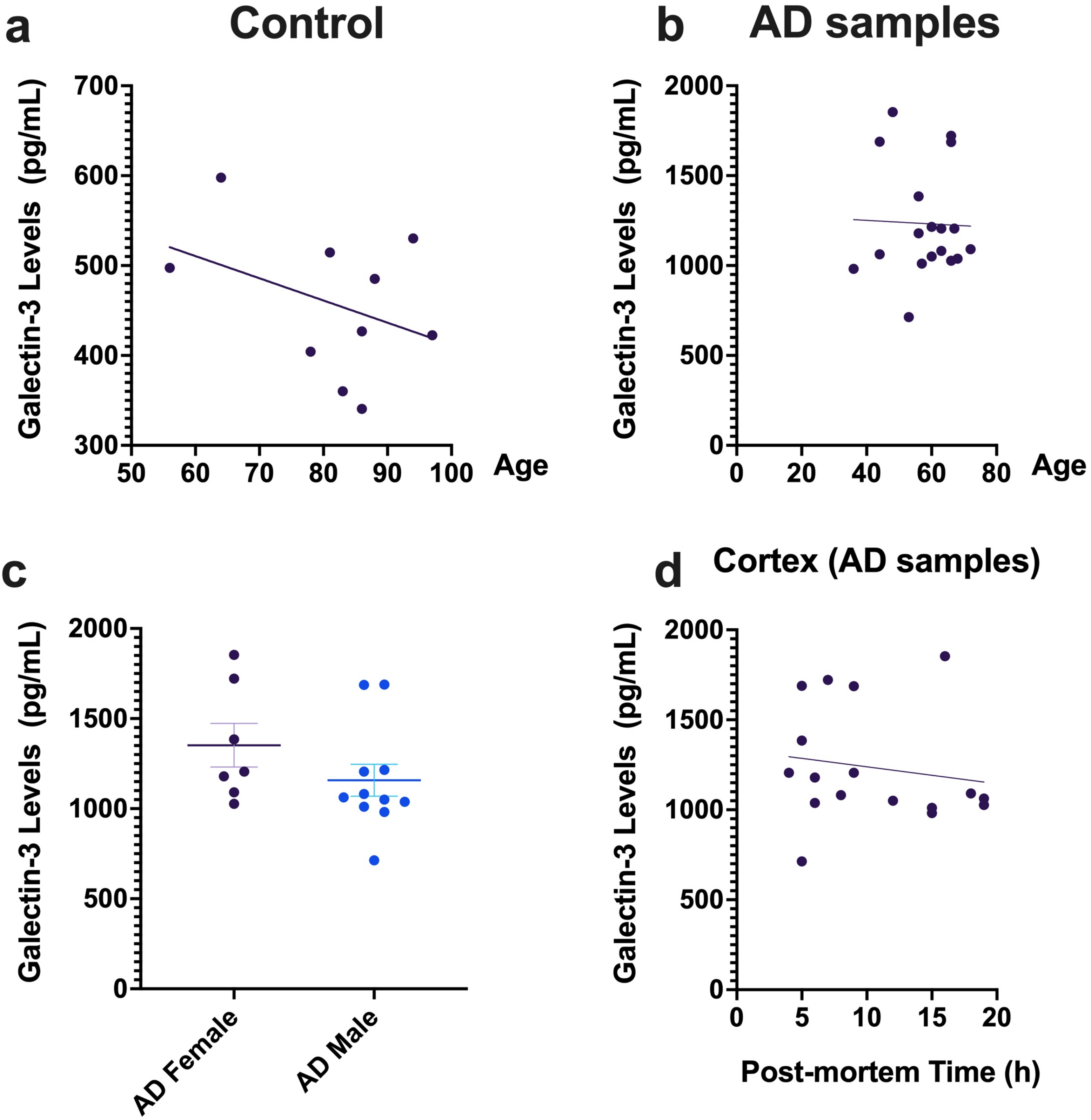

Supp. Figure 3

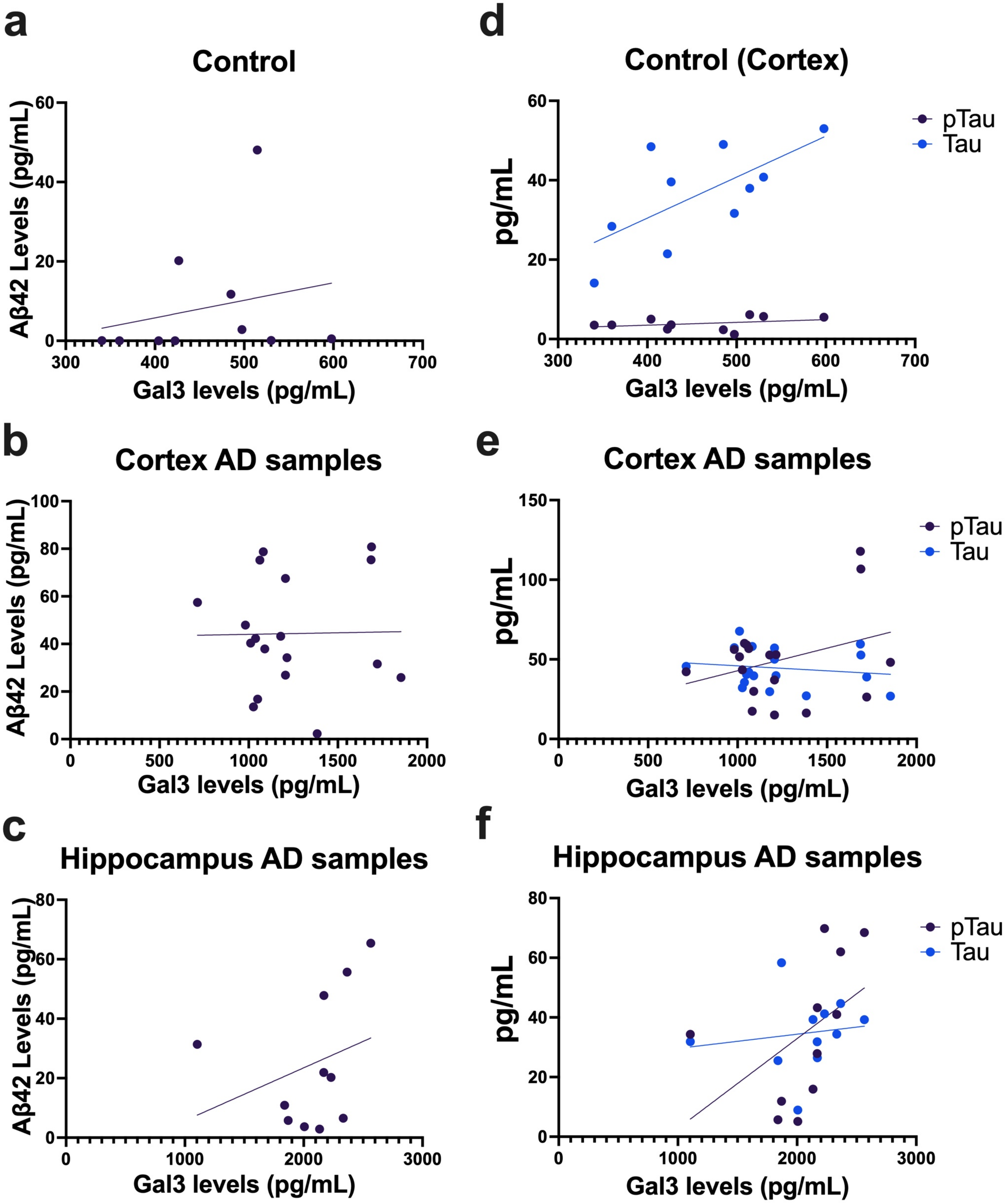

a

CSF Gal-3

n=155	NC	AD	
Predicted control	TN= 30	FN= 52	*n=82*
Predicted AD	FP= 6	TP= 67	*n=73*
	n=36	*n=119*	

b

CSF p-tau

n=155	NC	AD	
Predicted control	TN= 36	FN= 6	*n=42*
Predicted AD	FP= 0	TP= 113	*n=113*
	n=36	*n=119*	

c

CSF sTREM2

n=153	NC	AD	
Predicted control	TN= 23	FN= 46	*n=69*
Predicted AD	FP= 13	TP= 71	*n=84*
	n=36	*n=117*	

d

CSF tau

n=155	NC	AD	
Predicted control	TN= 35	FN= 14	*n=49*
Predicted AD	FP= 1	TP= 105	*n=106*
	n=36	*n=119*	

a

	ß	95% CI	P-value
**Whole cohort**	-0.042	-0.202 to 0.118	0.605
**NC**	0.010	-0.371 to 0.391	0.957
**AD**	**0.176**	**0.010 to 0.341**	**0.0217**

b

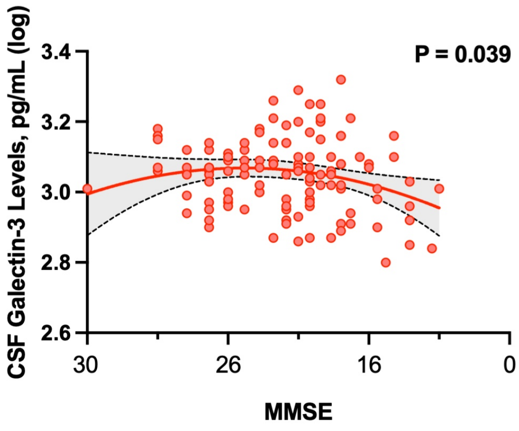

a

n = 97

	Component 1	Component 2
**Variance explained**	49 %	13%
**Eigen value**	4.4	1.2
**Loading values**		
gal-3		0.645
sTREM2		0.727
YKL-40		0.490
GFAP		0.845
Aß40/Aß42 ratio	0.685	
p-tau$_{181}$	0.797	
t-tau	0.802	
GAP-43	0.659	
neurogranin	0.785	

b

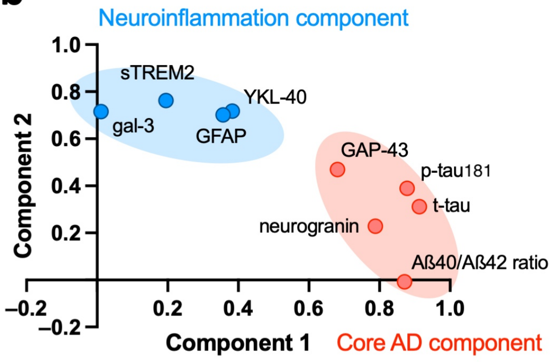

c

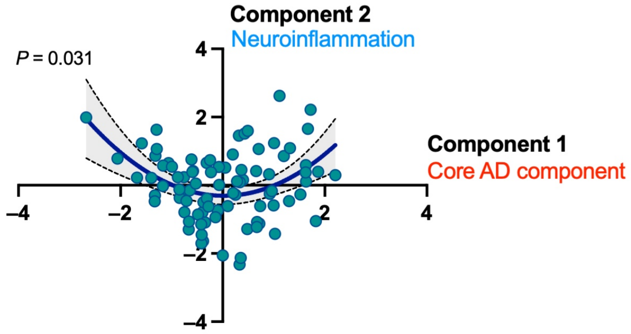

Supplement: Supplementary file 1 — Supplementary file1. Fig. 1. Aβ42 and p-Tau levels were upregulated in cortical and hippocampal samples from AD patients. a-b Aβ42 levels were significantly upregulated in cortical (AD-Cx) and hippocampal (AD-Hc) samples from AD patients compared to control samples. c-d) Total Tau levels were not significantly different between AD patients and controls in the cortex (AD-Cx) or hippocampus (AD-Hc). e–f) Phospho-Tau (p-Tau) levels were significantly upregulated in cortical (AD-Cx) and hippocampal (AD-Hc) samples from AD patients compared to control samples. g) Gal-3 levels from cortical samples from AD patients and controls were analyzed by western blot. Our data confirmed a clear up-regulation of Gal-3 in AD patients). Data are shown as Mean ± SEM. A parametric t-test was applied. *p < 0,05; **p < 0.01; ***p < 0.001; ****p < 0.0001. Fig. 2. Gal-3 levels are not affected by age, sex or post-mortem time in brain tissue from AD patients. a-d) Correlation analysis in control and AD brain samples was performed to evaluate whether age, sex, or post-mortem time induces an up-regulation of Gal-3. No significant correlation was found for any of the parameters evaluated. Simple regression analysis was performed in each case. Fig. 3. Aβ42 and tau levels do not correlate with Gal-3 levels in brain tissue of AD patients. a-c) Correlation analysis in cortex and hippocampus from controls and AD patients was performed. No significant differences were found in the analyses performed. c) A positive trend between Gal-3 and Aβ42 was observed in hippocampal samples from AD patients. Simple regression analysis was performed in each case. d-f) Correlation analysis in cortex and hippocampus from controls and AD patients were performed. No significant differences were found in any analysis performed. f) Positive trends between Gal-3 and Total Tau (Tau) and Gal-3 and p-Tau were found in hippocampal samples from AD patients. Simple regression analysis was performed in each case. Fig. 4. Confus [file 401_2022_2469_MOESM1_ESM.pdf]
