# Supplementary material for: Galectin-3 is elevated in CSF and is associated with Aβ deposits and tau aggregates in brain tissue in Alzheimer’s disease
Source: Acta Neuropathol. 2022 Jul 27;144(5):843–59. doi: 10.1007/s00401-022-02469-6 (PMC9547798; doi:10.1007/s00401-022-02469-6)
Supplement: Supplementary file 2 — Supplementary file2 (PDF 119 KB) [file 401_2022_2469_MOESM2_ESM.pdf]

Supplementary Table 1A (IDIBAPS)

Sample	Age	Sex	Neuropathological findings	Clinical Diagnose	PMD (hh:mm)	APOE
1	81	F	BRAAK 1, Thal 5, CERAD 1, CAA	Control	23:30	APOE 3/4
2	64	M	BRAAK II, Thal 2, CERAD A	Control	10:00	APOE 3/4
3	56	F	BRAAK I-II, Thal 1, CERAD none	Control	14:00	APOE 3/4
4	86	M	BRAAK III, Thal 0, Cerad None, moderate CAA	Control	07:25	Missing
5	78	M	BRAAK I-II, Thal 0, CERAD 0	Control	06:00	APOE 3/3
6	88	F	BRAAK II, Thal 3, CERAD moderate, moderate CAA	Control	24:00:00	Missing
7	83	F	Braak II, Thal 0, CERAD none	Control	07:30	Missing
8	97	F	BRAAK II, Thal 5, CERAD moderate, mild CAA	Control	07:20	Missing
9	86	M	Braak II, Thal3, Cerad B	Control	07:25	Missing
10	94	M	BRAAK II THAL 5 CERAD B + CAA	Control	15:46	Missing

Sample	Age	Sex	Neuropathological findings	Clinical Diagnose	PMD (hh:mm)	APOE
1	56	F	Braak V, Thal 5, , CERAD frequent	Familial Alzheimer's disease, PSEN1	05:00	APOE 3/3
2	54	M	Braak V, Thal 5, CERAD frequent	Familial Alzheimer's disease, PSEN1	07:30	Missing
3	57	M	THAL 5, BRAAK VI, CERAD FREQUENT, SEVERE CAA	Familial Alzheimer's disease, PSEN1	15:25	APOE 3/3
4	56	F	BRAAK VI, THAL 5, CERAD FREQUENT, SEVERE CAA	Familial Alzheimer's disease, PSEN1	06:00	APOE 4/4
5	44	M	BRAAK VI, THAL 5, CERAD FREQUENT, SEVERE CAA	Familial Alzheimer's disease, PSEN1	05:30	APOE 3/3
6	60	M	BRAAK VI, THAL 5, CERAD FREQUENT, SEVERE CAA	Familial Alzheimer's disease, PSEN1	07:25	APOE 3/4
7	53	M	BRAAK VI, THAL 5, CERAD FREQUENT, SEVERE CAA	Familial Alzheimer's disease, PSEN1	05:25	APOE 2/3
8	48	F	THAL 5, BRAAK VI, CERAD FREQUENT, SEVERE CAA	Familial Alzheimer's disease, PSEN1, onset 39-40y	16:40	Missing
9	44	M	Braak VI, THAL 5, CERAD frequent + posterior CAA	Familial Alzheimer's disease, PSEN1, onset 37y	19:33	APOE 3/4

10	36	M	Braak VI, Thal 5, CERAD frequent	Familial Alzheimer's disease, APP mutation, onset 30y	15:00	APOE 3/3
11	68	M	BRAAK V, THAL 5,CERAD FREQUENT + SEVERE CAA	Familial Alzheimer's disease, APP duplication, FT predominant	06:10	Missing
12	66	M	BRAAK VI, THAL 5, CERAD C	early-onset Alzheimer's disease, onset 55y	09:00	APOE 3/3
13	63	F	BRAAK VI, THAL 5, CERAD FREQUENT, MODERATE CAA	early-onset Alzheimer's disease	09:00	APOE 3/3
14	72	F	BRAAK VI, THAL 5, CERAD FREQUENT, SPARSE CAA	early-onset Alzheimer's disease, onset 62y	18:00	APOE 3/3
15	66	F	Braak VI, Thal 5, CERAD frequent	early-onset Alzheimer's disease, onset 51y	19:00	APOE 3/3
16	60	M	Braak VI, Thal 4-5, CERAD moderate	early-onset Alzheimer's disease, onset 51y	12:00	APOE 3/3
17	63	M	BRAAK VI, THAL 5, CERAD FREQUENT, SPARSE CAA	early-onset Alzheimer's disease, onset 56y	08:00	APOE 3/3
18	66	F	Braak VI, Thal 5, CERAD frequent, CAA	early-onset Alzheimer's disease, onset 57y, memory and language alteration	07:00	APOE 3/3

PMD: Post Mortem Delay

Supplementary Table 1B. The Netherlands BioBank

Sample	Clinical Diagnosis	Neuropath. evaluation<br>Aß / NFT / LB	Gender	Age	ApoE	PMD
1	Non-demented	Braak I / NFT zero / LB score 0	F	75	ApoE 3/3	05:25
2	Non-demented	Braak I / NFT some / LB score 0	F	72	ApoE 3/3	06:50
3	Non-demented	Braak I / NFT some / LB score 0	F	75	ApoE 3/2	09:10
4	Alzheimer's disease	Braak VI / NFT many / LB score 0	M	72	ApoE 3/4	05:15
5	Alzheimer's disease	Braak VI / NFT many / LB score 6	M	64	ApoE 3/4	08:15
6	Alzheimer's disease	Braak V / NFT many / LB score 0	M	82	ApoE 3/4	04:25
7	Alzheimer's disease	Braak III / NFT moderate / LB score 0	M	83	ApoE 3/4	06:40
8	Alzheimer's disease	Braak V / NFT moderate / LB score 0	M	94	ApoE 3/3	04:15
9	Alzheimer's disease	Braak V / NFT moderate / LB score 0	M	68	ApoE 3/3	09:15
10	Alzheimer's disease	Braak VI / NFT moderate / LB score 0	M	76	ApoE 4/4	07:32
11	Alzheimer's disease	Braak IV / NFT moderate / LB score 0	F	81	ApoE 3/4	08:10

PMD: Post Mortem Delay

Supplementary Table 1, Human post-mortem Neuropathological findings from IDIBAPS (1A) and The Netherlands Biobank* (1B)

*For the Netherlands Biobank. The presence of neurofibrillary tangles (NFTs) and neuropil threads (NTs) was scored according to Braak stages I-VI[2] and the Ab plaques were scored into O, A, B, and C (where O=zero, A=some, B=moderate, and C=many) according to Braak[2]. Lewy bodies was scored according to Braak stages 0-6[1].

1 Braak H, Bohl JR, Muller CM, Rub U, de Vos RA, Del Tredici K (2006) Stanley Fahn Lecture 2005: The staging procedure for the inclusion body pathology associated with sporadic Parkinson's disease reconsidered. Mov Disord 21: 2042-2051 Doi 10.1002/mds.21065

2 Braak H, Braak E (1991) Neuropathological stageing of Alzheimer-related changes. Acta Neuropathol 82: 239-259 Doi 10.1007/BF00308809

Supp. Table 2. Performance of each biomarkers cut-offs for AD identification

**Variables**	**Cut-off value**	**Sensitivity (Recall)**	**Specificity**	**NPV**	**PPV (Precision)**	**Accuracy**
Gal-3	> 1108 pg/mL	56%	83%	36%	92%	63%
sTREM2	> 3935 pg/mL	61%	63%	33%	85%	61%
p-tau	> 49.30 pg/mL	95%	100%	86%	100%	96%
total tau	> 371.5 pg/mL	88%	97%	71%	99%	90%

Sensitivity is calculated as TP/(TP + FN), specificity as TN/(TN + FP), and accuracy as (TP + TN)/(TP + FP + FN + TN) from the confusion matrix.
Abbreviations: AD, Alzheimer's disease; FP, false positive; FN, false negative; NPV, negative predictive value; PPV, predictive positive value; TP, true positive; TN, true negative

	n=125 patients with available CSF and plasma albumin measures	Neurological Controls* n = 19	AD* n = 106
**Albumin quotient**	**6.81 ± 3.41**	**6.44 ± 2.34**	**6.88 ± 3.57**
**CSF albumin, g/L**	**0.286 ± 1.151**	**0.276 ± 95.3**	**0.288 ± 159.7**
**Plasma albumin, g/L**	**42.1 ± 3..22)**	**43.2 ± 3.90**	**41.9 ± 3.06**
**Correlation of albumin markers with CSF glial and astrocytic biomarkers**			
**CSF Gal-3, pg/mL**	**Alb. quotient: rho=0.255, p=0.0043**<br>**CSF Alb.: rho=0.216, p= 0.0180**<br>Plasma Alb.: rho=-0.075, p= 0.4044	Alb. quotient: rho=0.188, p=0.4415<br>CSF Alb.: rho=0.2I7, p=0.3710<br>Plasma Alb.: rho=-0.181, p=0.4587	**Alb. quotient: rho=0.263, p=0.0067**<br>**CSF Alb.: rho=0.226, p=0.0231**<br>Plasma Alb.: rho=-0.049, p=0.6152
**CSF sTREM2, pg/mL**	**Alb. quotient: rho=0.209, p=0.019**<br>**CSF Alb.: rho=0.203, p=0.023**<br>Plasma Alb.: rho=-0.077, p=0.391	**Alb. quotient: rho=0.623, p=0.004**<br>**CSF Alb.: rho=0.646, p=0.003**<br>Plasma Alb.: rho=-0.123, p=0.616	Alb. quotient: rho=0.164, p=0.094<br>CSF Alb.: rho=0.164, p=0.093<br>Plasma Alb.: rho=-0.051, p=0.602
**CSF YKL-40, pg/mL**	Alb. quotient: rho=0.101, p=0.274<br>CSF Alb.: rho=0.079, p=0.388<br>Plasma Alb.: rho=-0.115, p=0.208	Alb. quotient: rho=0.298, p=0.229<br>CSF Alb.: rho=0.269, p=0.280<br>Plasma Alb.: rho=-0.221, p=0.378	Alb. quotient: rho=0.064, p=0.524<br>CSF Alb.: rho=0.050, p=0.619<br>Plasma Alb.: rho=-0.048, p=0.631
**CSF GFAP, pg/mL**	**Alb. quotient: rho=0.445, p<0.001**<br>**CSF Alb.: rho=0.425, p< 0.001**<br>Plasma Alb.: rho=-0.077, p=0.402	Alb. quotient: rho=0.211, p=0.415<br>CSF Alb.: rho=0.250, p=0.332<br>Plasma Alb.: rho=0.087, p=0.739	**Alb. quotient: rho=0.470, p<0.001**<br>**CSF Alb.: rho=0.458, p< 0.001**<br>Plasma Alb.: rho=-0.057, p=0.569

Supplementary Table 3, Association of CSF biomarkers of neuroninflammation with CSF and plasma albumin

*AD versus NC compared using Mann-Whitney test: albumin quotient, P= 0.8284; CSF albumin: P= 0.6647; plasma albumin: P= 0.2474.
Correlations between markers were studied using Spearman correlation.

Abbreviations: AD, Alzheimer disease; Alb., albumin; CSF, cerebrospinal; Gal-3, Galectin-3
